# Vinburnine Sensitizes Radiotherapy Efficacy in Nasopharyngeal Carcinoma by Triggering Pyroptosis and Immune Responses via Activation of EDAR‐NFκB Pathway

**DOI:** 10.1002/advs.202506139

**Published:** 2025-09-25

**Authors:** Jing Chen, Nian Liu, Qian Tao, Jie Wu, Jinyu Huang, Can Lu, Qiuqiu Li, Liangfang Shen, Xiang Chen, Cong Peng

**Affiliations:** ^1^ Department of Dermatology Xiangya Hospital Central South University Changsha Hunan 410000 China; ^2^ Department of Oncology Xiangya Hospital Central South University Changsha Hunan 410000 China; ^3^ Hunan Key Laboratory of Skin Cancer and Psoriasis Human Engineering Research Center of Skin Health and Disease Xiangya Hospital Central South University Changsha Hunan 410000 China; ^4^ Furong Laboratory Central South University Changsha Hunan 410000 China; ^5^ National Clinical Research Center for Geriatric Disorders Xiangya Hospital Central South University Changsha Hunan 410000 China

**Keywords:** EDAR, nasopharyngeal carcinoma, pyroptosis, vinburnine

## Abstract

Radiotherapy combined with chemotherapy is the traditional treatment for nasopharyngeal carcinoma (NPC); however, the side effects of these therapies restrict their clinical application; therefore, identifying appropriate alternative chemotherapy agents is a critical clinical challenge. In this study, an approved drug library is assessed and identified vinburnine as a promising alternative chemotherapy agent that sensitizes NPC to radiotherapy with lower toxicity than cisplatin. Mechanistically, vinburnine directly interacts with ectodysplasin A receptor (EDAR), a member of the Tumor Necrosis Factor (TNF) receptor superfamily, which activates the radiotherapy‐induced Nuclear Factor Kappa B (NFκB) signaling pathway. As a consequence, vinburnine enhances radiotherapy‐induces apoptosis in NPC cells, promotes Gasdermin E (GSDME)‐mediated pyroptosis, and increases the secretion of chemokine (C‐C motif) ligand 5(CCL5) and C‐X3‐C Motif Chemokine Ligand 1 (CX3CL1), which promotes and strengthens T‐cell toxicity against NPC cells. Furthermore, it is found that EDAR expression is significantly greater in patients with nonrecurrent NPC than in those with recurrent disease and that EDAR expression is positively correlated with CD8^+^ T‐cell infiltration; thus, it may be a potential biomarker for NPC prognosis. Overall, the study revealed that vinburnine is a novel chemotherapeutic agent that increases the sensitivity of NPC to radiotherapy and revealed a novel mechanism by which vinburnine and radiotherapy collaboratively modulate the EDAR‐NFκB‐apoptosis/pyroptosis‐CCL5/CX3CL1 signaling pathway, which provides a promising therapeutic strategy for NPC.

## Graphical abstract

This study demonstrates that vinburnine, an approved cerebrovascular drug, synergizes with radiotherapy in nasopharyngeal carcinoma (NPC) by modulating EDAR‐NFκB signaling through directly binding to EDAR, leading to triggering apoptosis/pyroptosis, and amplifying CCL5/CX3CL1‐driven T‐cell cytotoxicity. Notably, EDAR overexpression predicts improved survival and CD8^+^ T‐cell infiltration, which provides a novel chemo‐radiotherapy strategy in NPC clinical treatment.

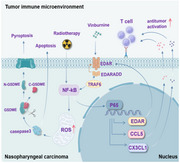

## 1 Introduction

Nasopharyngeal carcinoma typically responds well to radiotherapy, and the standard clinical approach involves chemoradiotherapy, with a 5‐year survival rate of ≈85%.^[^ ^1^ ^]^ However, chemoradiotherapy has inevitable side effects, including acute toxicities such as mucositis, dermatitis, xerostomia, and dysphagia, as well as late‐stage toxicities such as xerostomia, sensorineural hearing loss, osteoradionecrosis, trismus, central nervous system abnormalities, and hormonal dysfunction, which limit its clinical application.^[^ ^2^ ^]^ Additionally, 7.4% of patients experience recurrence or metastasis following conventional chemoradiotherapy; tumor recurrence and metastasis pose a severe threat to patients’ lives, accounting for 75% of NPC‐related deaths.^[^ ^3^ ^]^ Therefore, there is an urgent need for novel strategies to enhance the sensitivity of patients with NPC to radiotherapy, reduce radiation doses to minimize toxicity, and achieve optimal therapeutic outcomes.

Pyroptosis is a form of programmed cell death characterized by significant cellular responses to various stimuli, including inflammasome formation, maturation of inflammatory caspases, and caspase‐mediated cleavage of pyroptotic proteins. Pyroptosis plays an antitumor role in chemotherapy,^[^ ^4^, ^5^, ^6^, ^7^, ^8^, ^9^ ^]^ targeted therapy,^[^ ^10^ ^]^ and immunotherapy.^[^ ^11^ ^]^ Unlike apoptosis, pyroptosis involves cellular rupture, with large bubbles forming from the swollen cell membrane, releasing cellular contents, including various inflammatory molecules, which enhance immune cell infiltration and weaken the immunosuppressive effects of the tumor microenvironment, thus activating antitumor immunity.^[^ ^12^, ^13^ ^]^ For example, during pyroptosis in tumor cells, GSDME expression increases the phagocytosis of these cells by tumor‐associated macrophages. It also increases the quantity and cytotoxicity of infiltrating natural killer (NK) cells and CD8^+^ T lymphocytes, simultaneously triggering caspase‐independent pyroptosis in target cells by directly cleaving GSDME at a site analogous to caspase‐3.^[^ ^13^ ^]^ Pyroptosis also sensitizes cells to radiotherapy. GSDME expression increases the sensitivity of radiation‐resistant rectal cancer cells to radiation and induces GSDME‐mediated pyroptosis in CRC cells, enhancing antitumor immunity by promoting the recruitment and activation of NK cells.^[^ ^14^ ^]^ However, there are few reports on pyroptosis in NPC, and further studies are required to clarify its role in NPC.

Ectodysplasin A receptor (EDAR) is a prototypical member of the TNF receptor superfamily and contains a signal peptide, three cysteine‐rich domains (CRDs), a transmembrane domain, and an intracellular region containing the so‐called “death domain”.^[^ ^15^ ^]^ Similar to other TNF pathway components (such as TNF/TNFR/TRADD), EDAR activates downstream NFκB signaling by forming complexes with Ectodysplasin A receptor‐associated death domain protein (EDARADD) and TNF receptor‐associated factors (TRAFs).^[^ ^16^, ^17^ ^]^ Members of the TNF receptor family play critical roles in tumorigenesis by inducing apoptosis and periodic forms of cell death and regulating the survival, proliferation, differentiation, and effector functions of both immune and non‐immune cells.^[^ ^18^ ^]^ Although EDAR signaling primarily plays a role in embryonic ectodermal development,^[^ ^19^ ^]^ its antitumor activity and role in the immune response remain unclear.

In this study, we identified vinburnine as a novel radiosensitizer through screening of approved drugs, which increases the efficacy of radiotherapy for NPC by inducing cellular pyroptosis via the EDAR‐ NFκB pathway.

## 2 Results

### 2.1 Vinburnine Increases Radiotherapy Efficacy in NPC Cells

**Figure 1:**
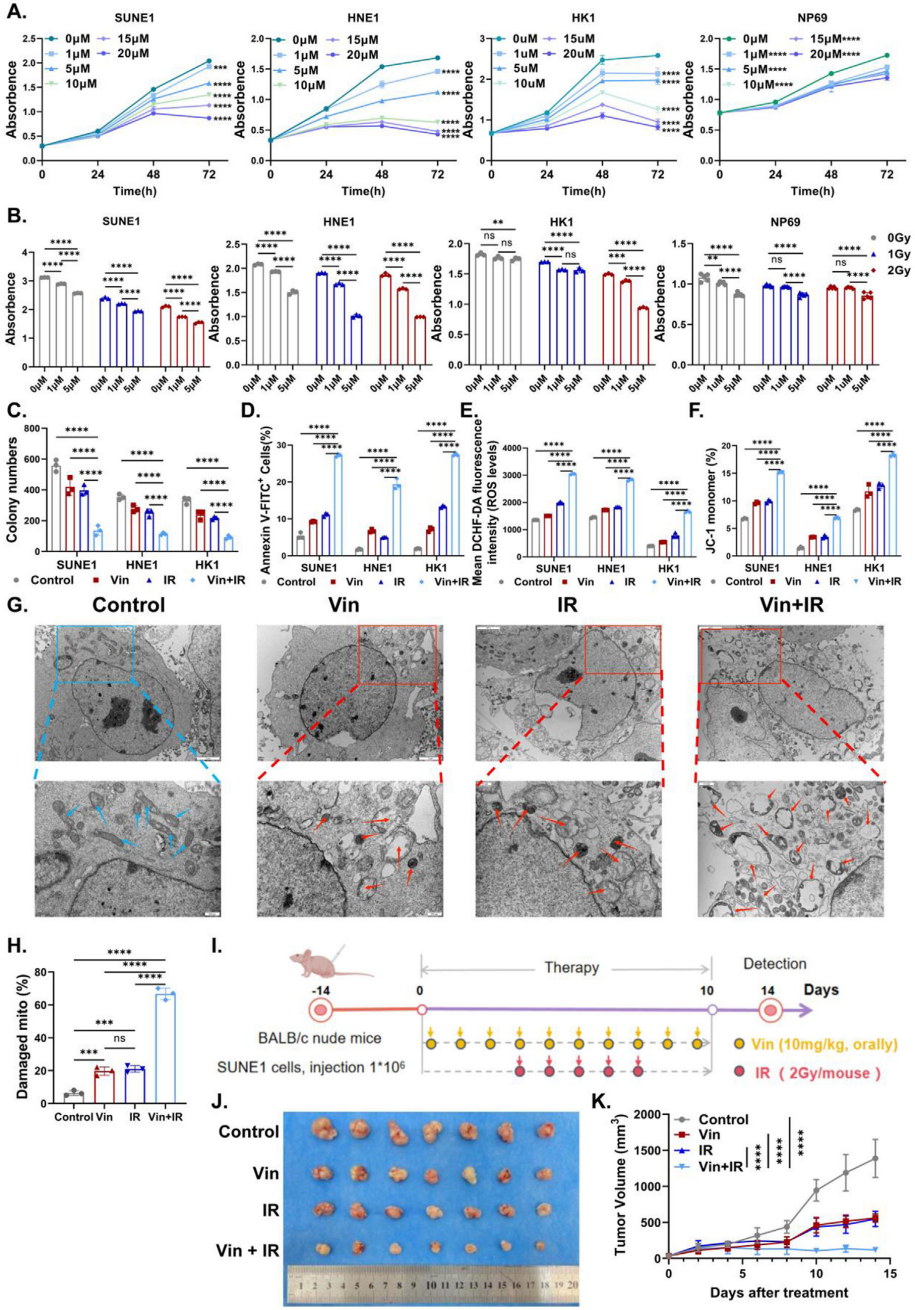
Vinburnine sensitizes radiPeriodicalapy efficacy in NPC cells. A) The cell viability of NPC cells (SUEN1/HNE1/HK1) and nasopharyngeal epithelial cells (NP69) treated with Vin (0–20µm) for 0–72h was measured by CCK8 assay (*n* = 5). B) The effects of Vin (1/5µm) combined with radiation (1/2Gy) on NPC cells (*n* = 3) and NP69 (*n* = 5) were detected by CCK8 for 48h. C) Detection of cell clone formation following treatment with 5µm Vin and 2Gy IR for 48h (*n* = 3). D) Flow cytometry was conducted to determine the apoptosis of NPC cells treated with 5µm Vin / 2Gy IR for 48h (*n* = 3). E) Flow cytometry was utilized to detect the ROS level in NPC cells following a 12 h treatment period (*n* = 3). F) Flow cytometry detected the mitochondrial membrane potential of NPC cells treated for 24h (*n* = 3). G, H) The mitochondrial damage of SUNE1 after treatment for 48h was detected and analyzed by electron microscopy (*n* = 3) (Scale bar: upper panel 2µm, lower panel 500nm. The blue arrows indicate normal‐shaped mitochondria, and the red arrows indicate autophagosomes). I) Schematic diagram of Vin combined with IR for NPC treatment. J, K) SUNE1 tumor‐bearing mice were treated with the indicated treatments to evaluate tumor volume effects (*n* = 7). Multiple samples were presented using mean ± standard deviation (SD). A, H, K) Statistical analysis with One‐way ANOVA was used to analyze the statistical differences among multiple groups. B–F) Statistical analysis with Two‐way ANOVA was used to analyze the statistical differences among multiple groups. ^*^ *p* < 0.05, ^**^ *p* < 0.01, ^***^ *p* < 0.001, ^****^ *p* < 0.0001, ns for non‐significant.

To identify potential radiosensitizing drugs, we initially screened a library of 2256 approved drugs for compounds that exhibit inhibitory effects on tumor cells.^[^ ^20^ ^]^ Building on this foundation, we selected five non‐oncology drugs with strong inhibitory effects for further screening of NPC cells. Among the non‐oncology drugs, we identified vinburnine (9317A7) as a compound that significantly inhibited the growth of NPC cells (Figure S1A, Supporting Information). To evaluate the cytotoxic effects of vinburnine on NPC tumors, we treated three types of NPC cells with varying concentrations for various durations. The results demonstrated that vinburnine exhibited time‐ and concentration‐dependent cytotoxicity against NPC cells, with IC50 values ranging from 8 to 16 µm (**Figure** **1**A; Figure S1B, Supporting Information). Furthermore, we investigated whether vinburnine increased the efficacy of radiotherapy. The results indicated a synergistic effect between vinburnine and radiotherapy, as the combination significantly enhanced tumor cell death (Figure 1B; Figure S1C, Supporting Information). We also determined the IC50 value of vinburnine and its radiosensitizing effect on normal nasopharyngeal epithelial cells (NP69). Notably, the IC50 value of vinburnine against NP69 cells was significantly greater than that against NPC cells, and no synergistic effect was observed when vinburnine was combined with radiotherapy to treat NP69 cells (Figure 1A,B; Figure S1B,C, Supporting Information). These findings suggest that vinburnine selectively targets NPC cells, indicating its potential as a safe radiosensitizer for NPC treatment.

Vinburnine also significantly suppressed the colony formation capacity of NPC cells when used in combination with radiation (Figure 1C; Figure S1D, Supporting Information). Flow cytometry analysis revealed that vinburnine combined with radiation (Vin+IR) significantly promoted the apoptosis of tumor cells (Figure 1D; Figure S1E, Supporting Information). To further explore the radiosensitizing effects of vinburnine, we assessed reactive oxygen species (ROS) production and mitochondrial membrane potential (according to JC‐1 staining), as both properties are related to radiosensitivity.^[^ ^21^, ^22^, ^23^, ^24^ ^]^ Vin+IR treatment markedly increased ROS levels and disrupted the mitochondrial membrane potential in NPC cells (Figure 1E,F; Figure S1F,G, Supporting Information). In addition, transmission electron microscopy confirmed severe mitochondrial damage in tumor cells treated with Vin+IR, with larger and rounder mitochondria, a shallower matrix, shorter and fewer cristae, and a significant increase in autophagosomes (red arrows) (Figure 1G,H). In vivo experiments using an NPC xenograft mouse model further validated the radiosensitizing effects of vinburnine (Figure 1I). The combination treatment significantly inhibited tumor growth (Figure 1J,K) without causing weight loss in mice, indicating a high safety profile of vinburnine (Figure S1H, Supporting Information). These findings demonstrated that vinburnine was a potent and safe radiosensitizer for the treatment of NPC.

### 2.2 Vinburnine Directly Binds to EDAR and Activates NFκB‐Mediated Apoptosis and Pyroptosis

**Figure 2:**
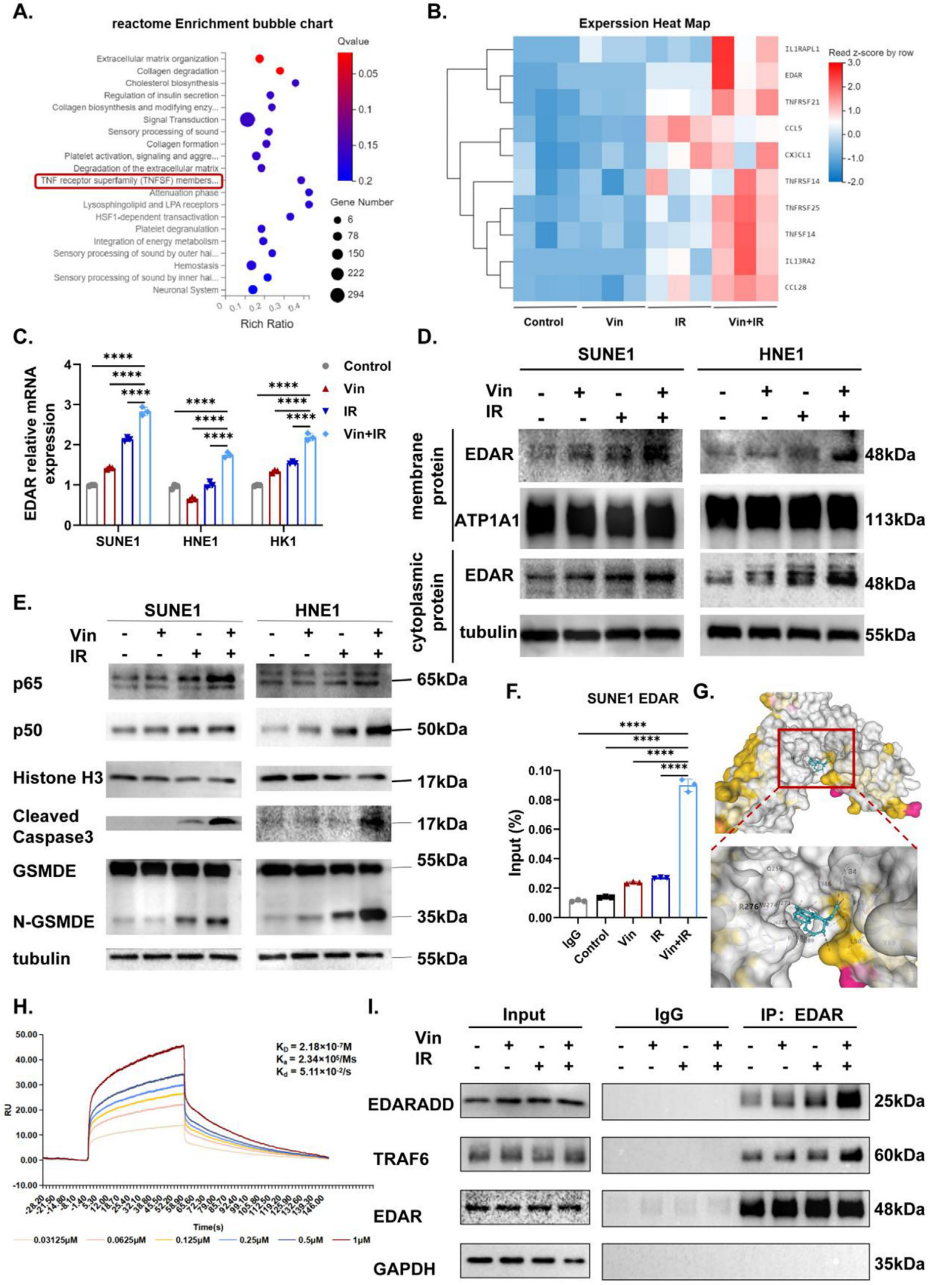
Vinburnine promotes IR‐EDAR‐NFκB‐induced apoptosis and pyroptosis. A) Reactome analysis of upregulated differential genes after 5µm Vin / 2Gy IR treatment for 48h. B) Heatmap of differential gene expression in transcriptomics. C) qPCR of EDAR mRNA in NPC cells treated with 5µm Vin / 2Gy IR for 48h (*n* = 3). D) Protein expression of EDAR in the cell membrane/cytoplasm after being treated with 5µm Vin / 2Gy IR for 48h. E) Western blotting detected the protein expression levels of the NFκB pathway (p65/p50) and pyroptosis index (GSDME/N‐GSDME/ Cleaved‐Caspase3) after 5µm Vin / 2Gy IR treatment for 48h. Upon activation of the NFκB signaling pathway, its downstream signal, Caspase3, undergoes cleavage. This cleavage then cuts GSDME to form N‐GSDME, ultimately resulting in the pyroptosis of the cells. F) The binding of p65 to the EDAR promoter in the treated SUNE1 cells was detected by ChIP assay. G) The CB‐Dock2 website predicts the structural complex of vinburnine bound with the EDAR protein. Vinburnine is colored green; EDAR is colored grey and yellow. H) SPR technology proved that EDAR is the target of vinburnine. I) The Co‐IP experiment confirmed that after treatment with 5µm Vin+2Gy IR, EDAR formed more protein complexes with EDARADD/TRAF6. Multiple samples were presented using mean ± standard deviation (SD). C) Statistical analysis with Two‐way ANOVA was used to analyze the statistical differences among multiple groups. F) Statistical analysis with One‐way ANOVA was used to analyze the statistical differences among multiple groups. ^*^ *p* < 0.05, ^**^ *p* < 0.01, ^***^ *p* < 0.001, ^****^ *p* < 0.0001, ns for non‐significant.

To elucidate the molecular mechanisms underlying the radiosensitizing effects of vinburnine, we conducted RNA‐seq analysis of the control, vinburnine, radiation, and combination treatment groups in SUNE1 cells. As shown in Figure S2A (Supporting Information), Vin+IR significantly altered gene expression in SUNE1 cells. We further analyzed the non‐overlapping differentially expressed genes (DEGs) between the combination and single agent groups, as this subset represented the unique transcriptional features induced by the combination treatment (Figure S2B, Supporting Information). Further analysis of these DEGs revealed that the combination of vinburnine and irradiation (IR) promoted TNF signaling in SUNE1 cells (**Figure** **2**A,B). However, our in vitro validation revealed that, among the upregulated TNF family members, only EDAR exhibited a significantly increased expression after Vin+IR treatment (Figure 2C; Figure S2C, Supporting Information). Moreover, Vin+IR significantly increased the protein levels in both the cell membrane and cytoplasm (Figure 2D; Figure S2D, Supporting Information). TNF signaling regulates MAPK, NFκB, mitochondrial apoptosis, and the immune response.^[^ ^25^ ^]^ Moreover, Vin+IR treatment promoted the formation of cleaved‐caspase 3, a downstream component of the NFκB signaling pathway^[^ ^26^ ^]^ (Figure 2E; Figure S2E, Supporting Information), leading to increased apoptosis (Figure 1D; Figure S1E, Supporting Information). Our data excluded MAPK activation as a contributing factor (Figure S2F, Supporting Information). These findings suggest that vinburnine amplifies IR‐induced NFκB‐EDAR signaling to promote NPC cell death. Further JASPAR transcription factor prediction revealed multiple p65 binding sites on the EDAR promoter (Figure S2G, Supporting Information). Primers were designed and ChIP experiments were performed based on the predicted binding sites, and it was found that p65 directly bound to the EDAR promoter at ‐1181 to ‐1164 bp, and Vin+IR enhanced the binding of p65 to the EDAR promoter (Figure 2F). These results suggest that vinburnine enhances IR‐induced NFκB‐EDAR signaling. However, the specific mechanism through which vinburnine increased radiosensitivity remains unclear. EDAR, a member of the TNF receptor family, localizes to the cell membrane. Upon activation by its ligand EDA, EDAR forms a complex with the adaptor proteins EDARADD and TRAF6, subsequently activating NFκB signaling.^[^ ^16^, ^27^, ^28^, ^29^ ^]^ Therefore, we hypothesized that vinburnine may positively regulate EDAR expression by binding to EDAR on the cell membrane, thereby promoting IR‐induced NFκB‐EDAR signaling. To test this hypothesis, we used CB‐DOCK2 to predict the binding sites between vinburnine and EDAR and found that vinburnine bound to EDAR through multiple sites (Figure 2G; Figure S2H, Supporting Information). Cellular thermal shift assays (CETSAs) revealed that EDAR was more stable in vinburnine‐pretreated samples than in control samples at various temperatures, confirming that EDAR was a binding target of vinburnine (Figure S2I, Supporting Information). Surface plasmon resonance (SPR) assays further validated the direct binding between vinburnine and EDAR, with a Kd value of 2.18 × 10^‐7 (Figure 2H). To validate the direct interaction between EDAR and its downstream adaptors EDARADD and TRAF6, we performed co‐immunoprecipitation (Co‐IP) assays using an anti‐EDAR antibody. The results showed that Vin+IR treatment markedly increased the expression of EDAR, EDARADD, and TRAF6 in the complex. Co‐IP assays confirmed that EDAR directly interacted with both EDARADD and TRAF6. These findings are consistent with previous reports,^[^ ^16^, ^27^, ^28^, ^29^ ^]^ supporting the conclusion that Vin+IR treatment activates downstream NF‐κB signaling by enhancing the formation of the EDAR–EDARADD–TRAF6 complex (Figure 2I; Figure S2J, Supporting Information). These results indicate that vinburnine directly binds EDAR to enhance IR‐induced NFκB‐EDAR positive feedback signaling.

Additionally, the percentage of apoptotic cells after vinburnine and IR treatment was less than 30% (Figure 1D; Figure S1E, Supporting Information), whereas the overall percentage of dead cells was 50% (Figure 1B). This finding led us to hypothesize that vinburnine, in combination with IR, may trigger an additional mode of cell death. Our results revealed that combination treatment induced GSDME‐mediated pyroptosis in tumor cells (Figure 2E; Figure S2E, Supporting Information). These results demonstrated that vinburnine functioned as a radiosensitizer by promoting IR‐induced EDAR expression and activating downstream NFκB signaling. Activated NFκB signaling induces apoptosis and pyroptosis and further enhances EDAR expression, creating a positive feedback loop that amplifies the EDAR‒NFκB axis.

### 2.3 Knockdown of EDAR Reverses the Radiosensitizing Effect of Vinburnine

**Figure 3:**
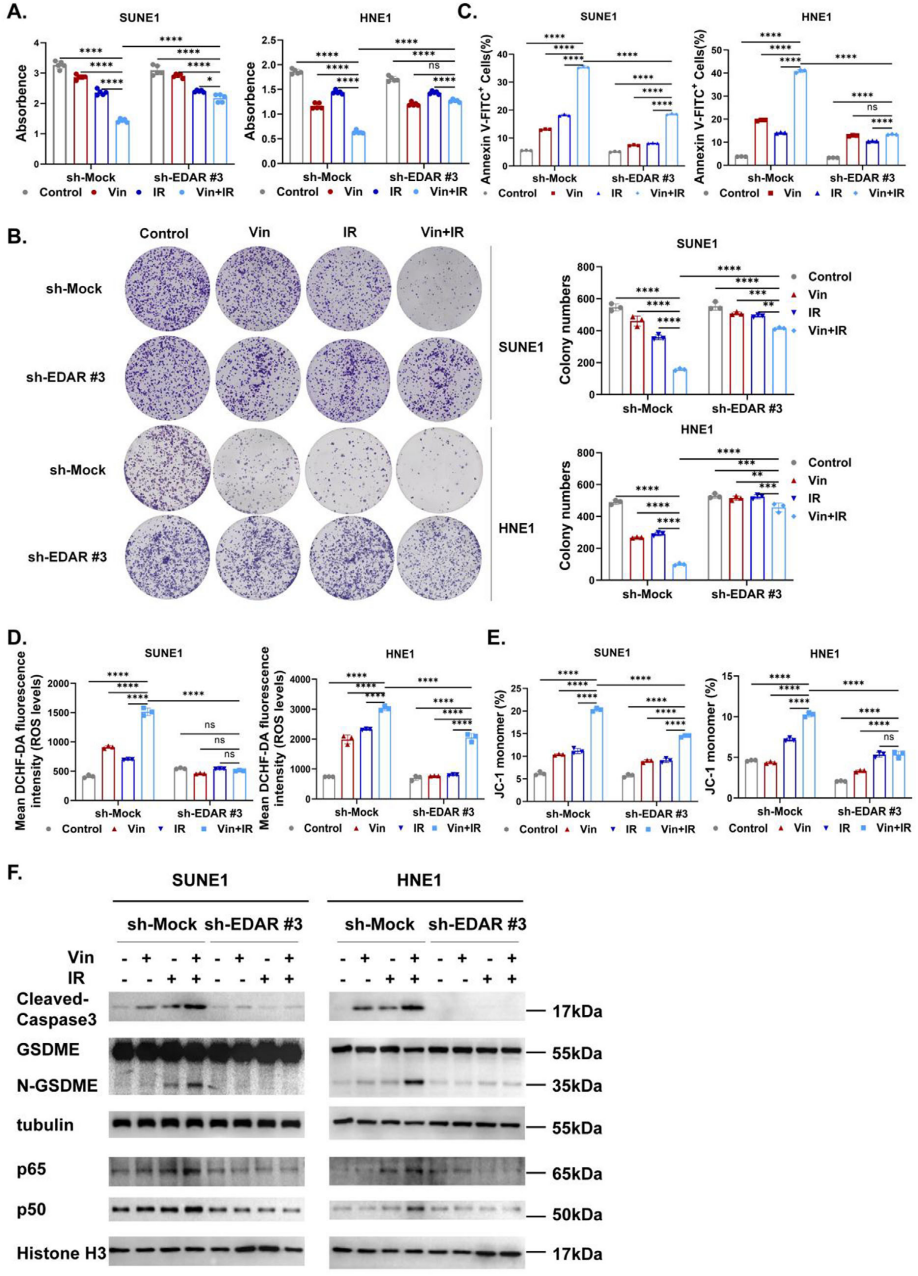
Knocking down EDAR suppresses the radiosensitization effect of vinburnine. A) The cytotoxic effect of 5uM Vin/2Gy IR on EDAR‐knockdown cells was assessed using the CCK‐8 assay (*n* = 5). B) Following EDAR knockdown, cells were treated with 5uM Vin/2Gy IR. Colony formation was evaluated by crystal violet staining (left) and the number of colonies was quantified (right) (*n* = 3). C–E) Flow cytometry detected the apoptosis/ROS levels/mitochondrial membrane potential of the Vin±IR‐treated cells after EDAR knockdown (*n* = 3). F) After EDAR knockdown, western blotting detected the protein expression (p65/p50/GSDME/N‐GSDME/Cleaved‐Caspase3) in the treated group. Multiple samples were presented using mean ± standard deviation (SD). A–E) Statistical analysis with Two‐way ANOVA was used to analyze the statistical differences among multiple groups. ^*^ *p* < 0.05, ^**^ *p* < 0.01, ^***^ *p* < 0.001, ^****^ *p* < 0.0001, ns for non‐significant.

To confirm that vinburnine increases radiosensitivity by promoting IR‐induced EDAR expression and activating downstream NFκB signaling, we knocked down EDAR expression in NPC cells (Figure S3A, Supporting Information). EDAR knockdown (shEDAR) did not affect NPC cell growth (Figure S3B, Supporting Information). However, after EDAR knockdown, the synergistic effect of vinburnine and IR was abolished, and the growth of NPC cells in the combination treatment group was partially recovered (**Figure** **3**A,B). Similarly, apoptosis in the combination group was significantly suppressed after EDAR knockdown (Figure 3C; Figure S3C, Supporting Information). Furthermore, ROS levels and mitochondrial membrane potential (Figure 3D,E; Figure S3D, Supporting Information) in the combination group were partially restored after shEDAR transfection. The knockdown of EDAR decreased the expression of cleaved caspase‐3, N‐GSDME, and the NFκB pathway components p65/p50 (Figure 3F; Figure S3E, Supporting Information). These results indicated that EDAR was a target of vinburnine and mediated its radiosensitizing effect.

### 2.4 Inhibition of NFκB Signaling Diminishes the Radiosensitizing Effect of Vinburnine

**Figure 4:**
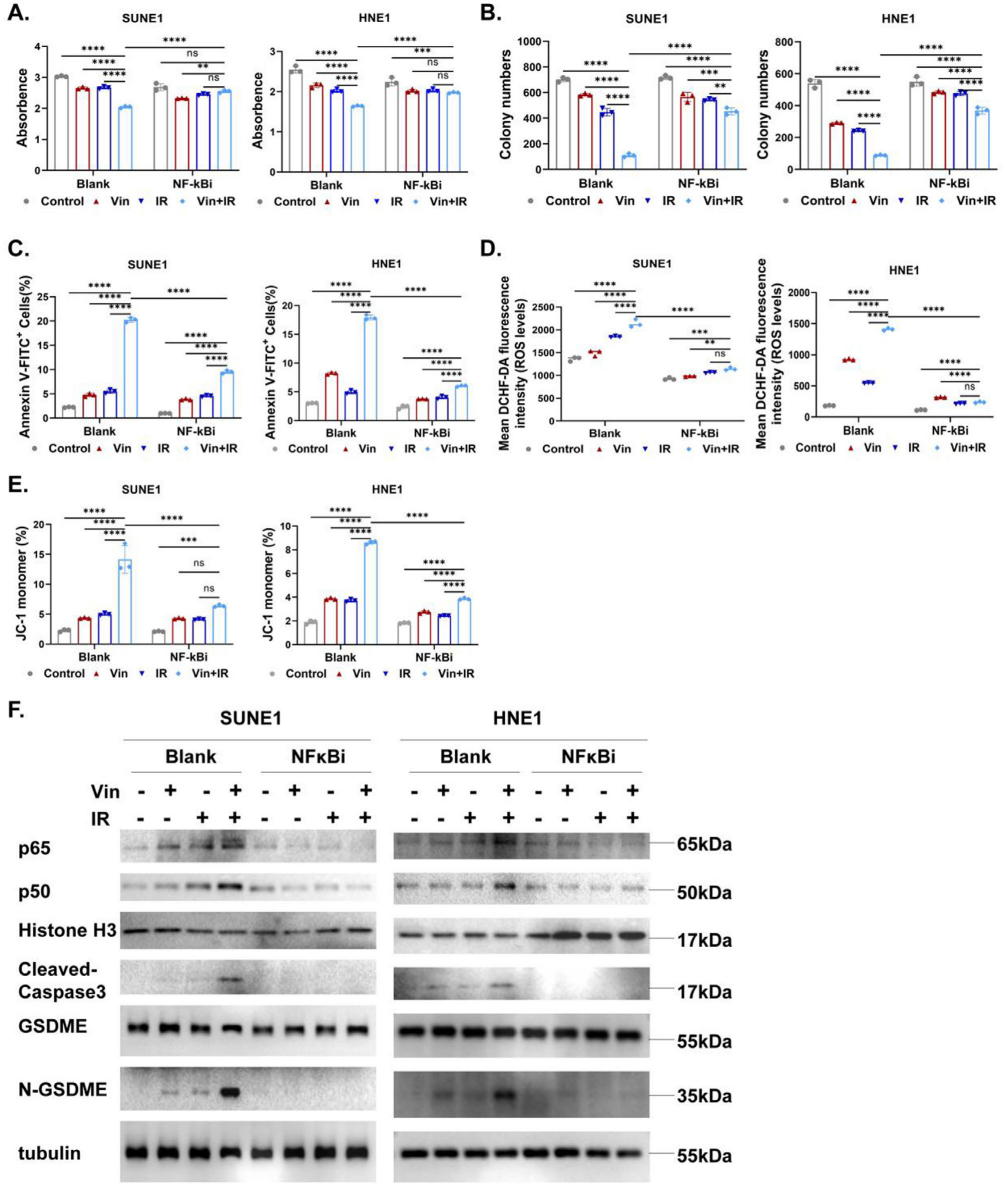
NFκB inhibitor suppresses the radiosensitizing effect of vinburnine. A) After pretreatment with 10µm NFκBi for 24h, the cell viability was assessed following treatment with 5µm Vin combined with 2Gy IR for 48h (*n* = 3). B) Following NFκBi pretreatment, the colony formation assay was utilized to evaluate and quantify the number of NPC cells under the 5µm Vin/2Gy IR treatment conditions (*n* = 3). C–E) Flow cytometry detected the apoptosis, ROS levels, and mitochondrial membrane potential of the Vin±IR‐treated cells following NFκBi pretreatment (*n* = 3). F) The expression of proteins (p65/p50/GSDME/N‐GSDME/Cleaved‐Caspase3) in the Vin/ IR‐treated cells was detected by western blotting following NFκBi pretreatment. Multiple samples were presented using mean ± standard deviation (SD). A–E) Statistical analysis with Two‐way ANOVA was used to analyze the statistical differences among multiple groups. ^*^ *p* < 0.05, ^**^ *p* < 0.01, ^***^ *p* < 0.001, ^****^ *p* < 0.0001, ns for non‐significant.

We next investigated whether vinburnine combined with IR induces mitochondrial damage and cell death via the NFκB signaling pathway. JSH‐23, an NFκB inhibitor, inhibits the transcriptional activity of NFκB by suppressing the nuclear translocation of NFκB p65.^[^ ^30^ ^]^ NPC cells were pretreated with an NFκB inhibitor (NFκBi) for 24 h and then treated with vinburnine and IR. As shown in **Figure** **4**A–C and S4A,B (Supporting Information), the radiosensitizing effect of vinburnine was abolished after NFκBi treatment (Figure 4A–C; Figure S4A,B, Supporting Information). Additionally, ROS levels and the mitochondrial membrane potential were restored in cells pretreated with the NFκB inhibitor (Figure 4D,E; Figure S4C, Supporting Information). The protein expression of cleaved caspase‐3, N‐GSDME, and p65/p50 decreased following NFκB inhibitor pretreatment (Figure 4F; Figure S4D, Supporting Information). These findings confirmed that the NFκB pathway was involved in the radiosensitizing effect of vinburnine, and that vinburnine combined with IR exerted its radiosensitizing effect through EDAR‐NFκB signaling.

### 2.5 Vin+IR Activates T Cell Immunity Through the EDAR‐NFκB‐CCL5/CX3CL1 Pathway

**Figure 5:**
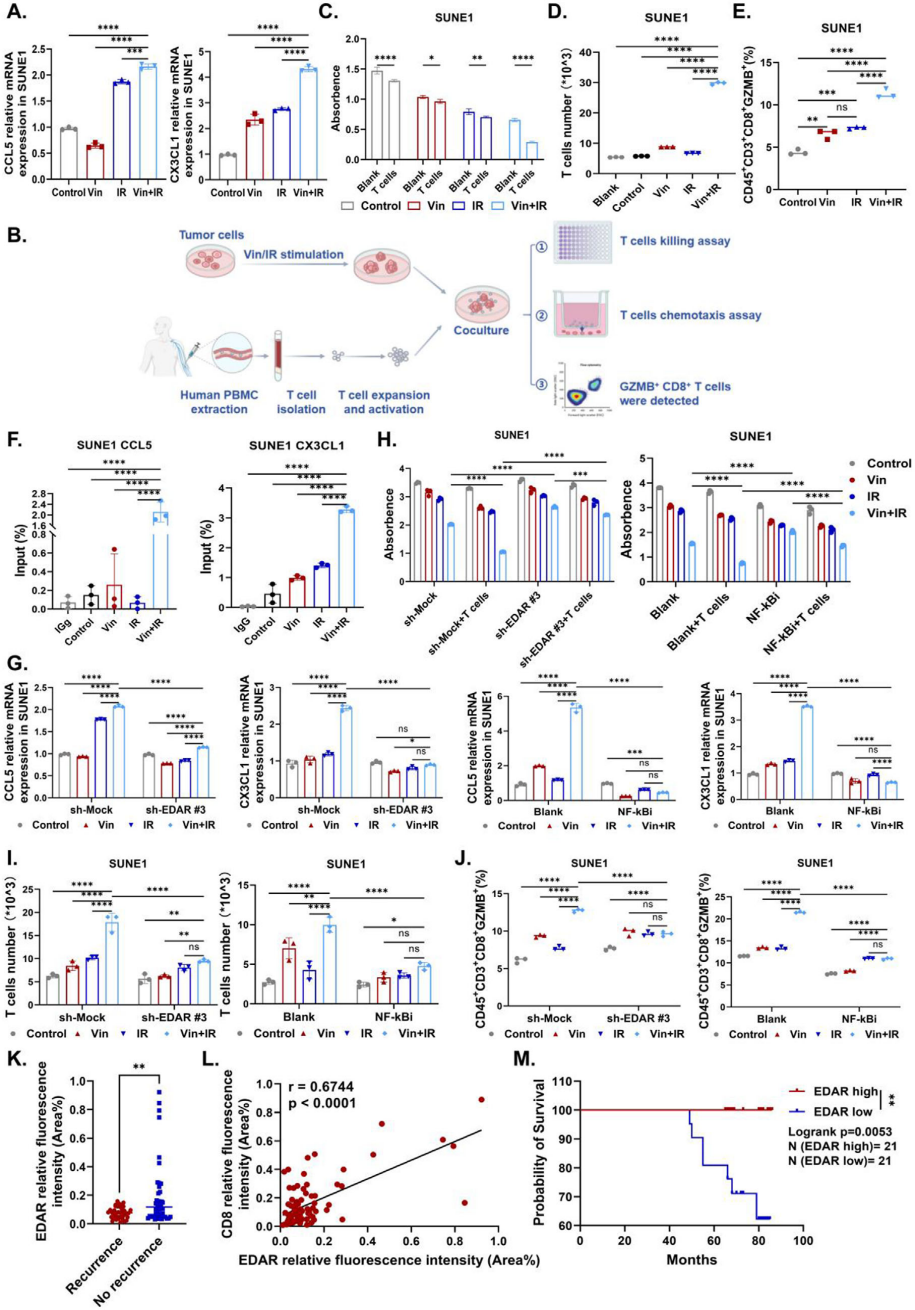
Vin+IR activates T cell immunity through the EDAR‐NFκB‐CCL5/CX3CL1 pathway. A) The mRNA expression of CCL5 and CX3CL1 in SUNE1 after 5µm Vin/ 2Gy IR treatment (*n* = 3). B) Diagram of human T cell‐related experiments. C). After 24h of 5µm Vin / 2Gy IR treatment, 1×10^4 SUNE1 was co‐cultured with 2.5×10^4 T cells for 48h. The suspended T cells were washed away with PBS, and the viability of tumor cells was detected by CCK8 (*n* = 5). D) T‐cell chemotaxis assay. The supernatant from SUNE1 cells treated with Vin/IR was collected and placed in the lower chamber of the transwell. T cells were then placed in the upper chamber of the transwell. After 48h, the liquid from the lower chamber was collected for T‐cell counting (*n* = 3). E) T cells were co‐cultured with treated SUNE1 cell supernatant for 48h, and CD45^+^CD3^+^CD8^+^GZMB^+^ T cells were detected by flow cytometry (*n* = 3). F) The ChIP assay was conducted to detect the binding of p65 to the CCL5 and CX3CL1 promoters following treatment with 5µm Vin / 2Gy IR for 48h (*n* = 3). G) Following sh‐EDAR or NFκBi pretreatment, the mRNA expression of CCL5 and CX3CL1 in Vin/IR‐treated SUNE1 was detected using qRT‐PCR (*n* = 3). H–J) After sh‐EDAR or NFκBi pretreatment, the proliferation of SUNE1 cells treated with 5µm Vin / 2Gy IR and co‐cultured with T cells, the number of chemotactic T cells in the supernatant of Vin/IR‐treated SUNE1 cells, and the proportion of CD45^+^CD3^+^CD8^+^GZMB^+^ T cells were measured (*n* = 3). K) EDAR and CD8 multiplex immunofluorescence staining was performed on nasopharyngeal carcinoma tissue microarrays. Comparison of mean fluorescence intensity of EDAR in tissues with recurrence (*n* = 39) and without recurrence (*n* = 45) in NPC patients. L) Correlation between EDAR and CD8 expression in human NPC tissues (*n* = 84). M) The patients were divided into EDAR low‐expression (*n* = 21) and EDAR high‐expression (*n* = 21) groups based on the quartile method. Survival analysis evaluated the association between EDAR expression levels and patient survival. Multiple samples were presented using mean ± standard deviation (SD). A, D–F) Statistical analysis with One‐way ANOVA was used to analyze the statistical differences among multiple groups. C, G–J) Statistical analysis with Two‐way ANOVA was used to analyze the statistical differences among multiple groups. K) The Mann‐Whitney test was used to analyze the expression of EDAR in patients with recurrence and those without recurrence. L) The correlation between EDAR and CD8 expression in tissue microarrays was analyzed using Pearson's and simple linear regression. M) Survival analysis (Kaplan‐Meier) was utilized to examine the correlation between EDAR expression and the prognosis of patients diagnosed with nasopharyngeal carcinoma. ^*^ *p* < 0.05, ^**^ *p* < 0.01, ^***^ *p* < 0.001, ^****^ *p* < 0.0001, ns for non‐significant.

Transcriptomic analysis revealed elevated cytokine signaling in the combination group, with a particularly pronounced increase in CCL5 and CX3CL1 expression (Figure 2B). The TNF signaling pathway is a complex biological cascade involving chemokines, cytokines, and endothelial adhesion molecules. This pathway can promote the recruitment and activation of lymphocytes at sites of injury or infection, thereby modulating the antitumor immune response.^[^ ^25^ ^]^ TNFα signaling induces NFκB p65 nuclear translocation, which promotes the transcription of CCL5 and CXC3L1, leading to their upregulation.^[^ ^31^, ^32^ ^]^ Moreover, CCL5 and CX3CL1 can recruit and activate T cell immunity.^[^ ^33^, ^34^, ^35^, ^36^, ^37^, ^38^, ^39^ ^]^ Additionally, radiotherapy enhances the secretion of T cells and T cell‐attracting chemokines, thereby boosting T cell‐mediated tumor killing.^[^ ^40^, ^41^ ^]^ The presence of TNF also sensitizes tumor cells to radiotherapy, thereby improving overall treatment.^[^ ^42^, ^43^, ^44^ ^]^ Therefore, we hypothesized that, as a member of the TNF superfamily, EDAR might regulate the expression of CCL5 and CX3CL1 via the NFκB pathway. We propose that the combination of vinburnine and IR activates the EDAR‐NFκB signaling pathway, promoting the expression and release of CCL5 and CX3CL1 to induce the T‐cell‐mediated immune response. First, we confirmed that the expression of CCL5 and CX3CL1 increased significantly in NPC cells after vinburnine treatment in combination with radiotherapy (**Figure** **5**A; Figure S5A, Supporting Information). Furthermore, our co‐culture experiments showed that Vin+IR treatment enhanced the T cell‐mediated killing of NPC cells (Figure 5C; Figure S5B, Supporting Information). Furthermore, using a Transwell system to separate the tumor supernatant from T cells, we found that the Vin+IR‐treated tumor supernatant increased T cell chemotaxis (Figure 5D; Figure S5C, Supporting Information). To assess whether the population of effector T cells had increased, we used flow cytometry with antibodies against CD45, CD3, and CD8 to identify CD45^+^CD3^+^CD8^+^ T cells.^[^ ^45^, ^46^, ^47^ ^]^ We then measured the proportion of Granzyme B⁺ CD8⁺ effector T cells after Vin+IR treatment, which is indicative of enhanced cytotoxic function.^[^ ^48^ ^]^ The results indicated that the proportion of functional T cells increased in the combination group (Figure 5E; Figure S5D, Supporting Information). Using the JASPAR software, we predicted multiple p65 binding sites in the promoter regions of CCL5 and CX3CL1. ChIP experiments confirmed the direct binding of P65 to the promoter regions of CCL5 (‐335 to ‐318 bp) and CX3CL1 (‐996 to ‐977 bp), and this binding was further enhanced by Vin+IR (Figure 5F; Figure S5E, Supporting Information). Knockdown of EDAR or pretreatment with an NFκB inhibitor reduced the expression of CCL5 and CX3CL1, further confirming that the EDAR‐NFκB pathway regulated CCL5 and CX3CL1 expression (Figure 5G; Figure S5F, Supporting Information). Additionally, both EDAR knockdown and NFκB inhibitor pretreatment weakened T cell cytotoxicity against NPC cells (Figure 5H; Figure S5G, Supporting Information), reduced the number of T cells recruited by tumor cell supernatants (Figure 5I; Figure S5H, Supporting Information), and decreased the proportion of GZMB^+^CD8^+^ T cells in the supernatant‐treated T cell population (Figure 5J; Figure S5I, Supporting Information). These findings demonstrated that vinburnine combined with radiotherapy induced the T cell‐mediated immune response by inducing the EDAR‐NFκB‐CCL5/CX3CL1 signaling pathway.

Next, we examined the correlation between EDAR and CD8 expression in NPC clinical tissue microarrays using multiplex immunofluorescence staining. The microarray analysis included 84 samples (39 recurrent cases and 45 non‐recurrent cases). All samples were confirmed to be NPC tissues by H&E staining (Figure S6A, Supporting Information). The results revealed that EDAR was highly expressed in patients without recurrence (Figure 5K; Figure S6B,C, Supporting Information). We also examined EDAR expression in tumor tissues from recurrent (*n* = 5) and non‐recurrent (*n* = 5) patients with NPC using IHC (Figure S6D, Table S5, Supporting Information). The results showed significantly higher EDAR expression in non‐recurrent cases, which is consistent with our tissue microarray findings (Figure S6E, Supporting Information). Additionally, EDAR expression was positively correlated with CD8 expression (Figure 5L). Survival analysis revealed that patients with high EDAR expression had significantly longer survival than those with low EDAR expression (Figure 5M). These findings suggest that the EDAR may be a prognostic biomarker for nasopharyngeal carcinoma.

### 2.6 Comparison of Tumor Suppressive Effects of Vin+IR+Hu‐T and Cis+IR+Hu‐T In Vivo

**Figure 6:**
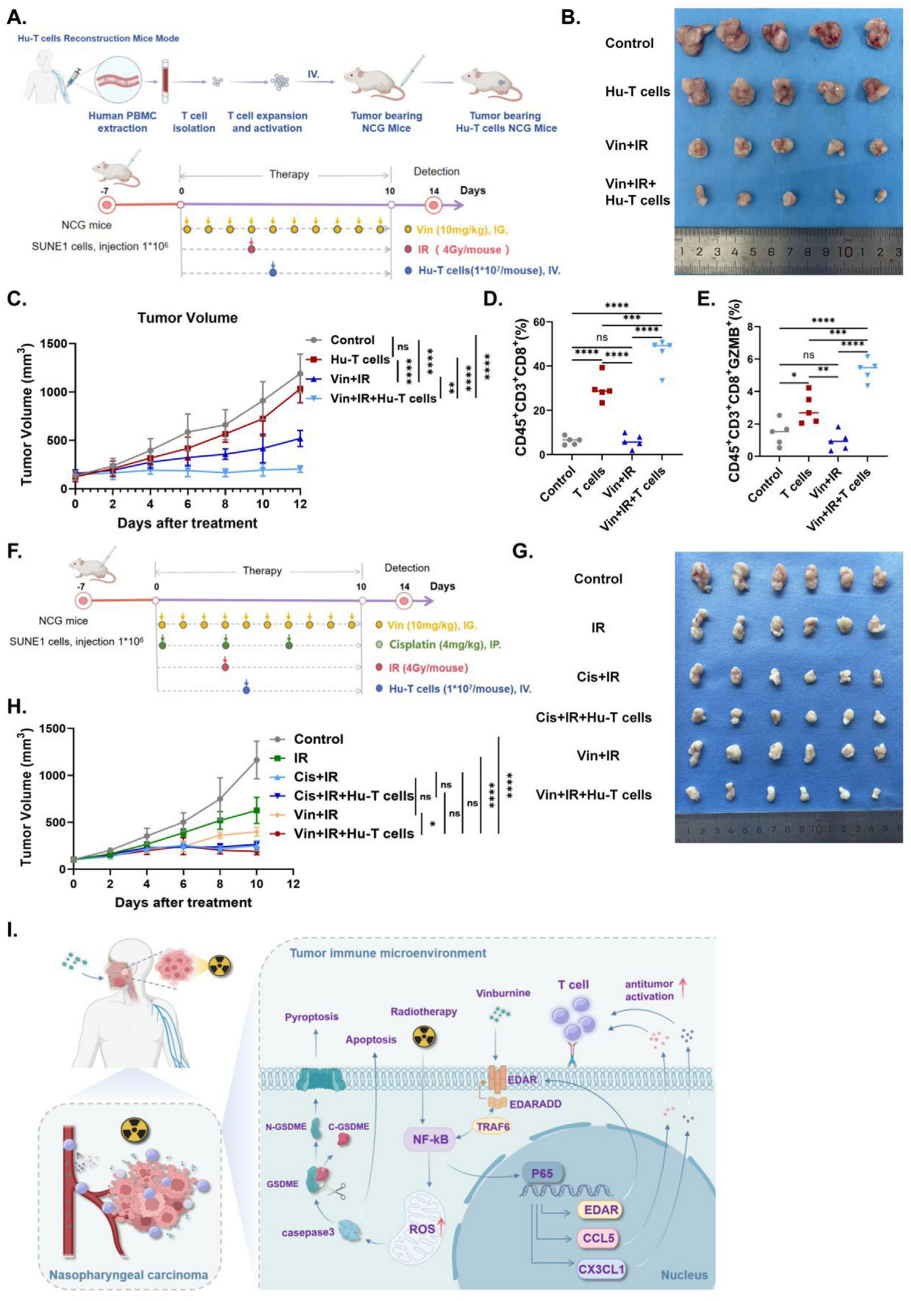
Comparison of tumor suppressive effects of Vin+IR+Hu‐T and Cis+IR+Hu‐T in vivo. A) Schematic diagram of animal experiments on an adoptive transfer of mice. B) Tumor tissues were isolated at the end of the experiment (*n* = 5). C) Tumor growth curves of SUNE1‐bearing mice with the indicated treatments (*n* = 5). D, E) Flow cytometry detected the proportion of CD45^+^CD3^+^CD8^+^ T cells and CD45^+^CD3^+^CD8^+^GZMB^+^ T cells in tumor tissue (*n* = 5). F) Schematic diagram of Cis/Vin combined radiPeriodicalapy in the treatment of NPC tumor‐bearing mice. G) Tumor tissues were isolated at the end of the experiment (*n* = 6). H) Tumor growth curves of SUNE1‐bearing mice with the indicated treatments (*n* = 6). I) Schematic diagram of the molecular mechanism of vinburnine combined with radiPeriodicalapy in the treatment of NPC. Multiple samples were presented using mean ± standard deviation (SD). C–E, H) Statistical analysis with One‐way ANOVA was used to analyze the statistical differences among multiple groups. ^*^ *p* < 0.05, ^**^ *p* < 0.01, ^***^ *p* < 0.001, ^****^ *p* < 0.0001, ns for non‐significant.

In vitro experiments demonstrated that the combination of vinburnine and radiotherapy significantly enhanced T cell activation. We constructed an adoptive transfer mouse model to validate this effect in vivo (**Figure** **6**A). As shown in Figure 6B,C, the injection of human T cells alone did not significantly control tumor growth. Moreover, Vin+IR+Hu‐T markedly inhibited tumor growth (Figure 6B,C). Notably, vinburnine treatment did not lead to further weight loss in the mice (Figure S7A, Supporting Information). Further analysis of the tumor immune microenvironment revealed a significant increase in the proportion of CD8^+^ T cells and exceptionally functional GZMB^+^CD8^+^T cells in the Vin+IR+Hu‐T group (Figure 6D,E). These results indicated that Vin+IR effectively killed NPC tumors in vivo by enhancing T cell function.

Currently, the recommended treatment regimen for locally advanced NPC involves three cycles of induction chemotherapy followed by concurrent chemoradiotherapy^[^ ^49^ ^]^ (Figure S7B, Supporting Information). Cisplatin plays a crucial role as a cornerstone drug in NPC treatment; however, its use is associated with significant adverse effects. As shown in Figure S7C (Supporting Information), cisplatin exhibited potent cytotoxicity against NPC cells and caused substantial damage to normal epithelial NP69 cells (Figure S7C, Supporting Information). Furthermore, a comparison of the combination index (CI) of Cis+IR and Vin+IR at equivalent 1/2 IC50 doses revealed that the CI of Cis+IR was not superior to that of Vin+IR (Figure S7D,E, Supporting Information). Additionally, in vivo experiments demonstrated that Vin+IR was as effective as Cis+IR in suppressing NPC tumor growth (Figure 6F,H). Notably, unlike the combination of Vin+IR, T cells did not affect the efficacy of Cis+IR treatment. These results suggest that the radiosensitizing effect of cisplatin is more likely to result from its cytotoxic properties rather than its ability to enhance the immune response. Additionally, mice treated with cisplatin experienced significant weight loss during the treatment period, highlighting the severe side effects of cisplatin (Figure S7F, Supporting Information). These findings indicate that vinburnine is a novel, safe, and effective radiosensitizer that increases radiotherapy efficacy through immune activation with minimal side effects.

On the basis of these findings, our study demonstrated that vinburnine promoted the IR‐induced NFκB‐EDAR‐CCL5/CX3CL1 signaling pathway. This activation induced mitochondrial damage and ROS accumulation in tumor cells, leading to apoptosis and pyroptosis of NPC cells. Moreover, the activation of NFκB signaling also enhanced the T‐cell immune response by promoting the expression of chemokines CCL5 and CX3CL1 (Figure 6I).

## 3 Discussion

Radiotherapy is a fundamental treatment approach for malignant tumors and is used in nearly all curative and palliative strategies for solid tumors. However, ionizing radiation can damage normal tissues, leading to acute and late toxicity. Consequently, a major challenge in radiotherapy is to enhance the damage to tumor tissues while minimizing the adverse effects on healthy tissues. An effective strategy for addressing this challenge is the incorporation of radiosensitizers. These approaches increase the effectiveness of radiotherapy, allowing for a decrease in the required radiation dose while reducing collateral damage to healthy tissues. Currently, there is a limited selection of clinically applicable radiosensitizers. Conventional agents such as sodium glycididazole result in only modest improvements in sensitivity,^[^ ^50^ ^]^ whereas chemotherapeutic agents such as cisplatin markedly increase the efficacy of radiotherapy. However, because of the severe side effects of chemotherapy, careful dosage management is often needed.^[^ ^51^ ^]^ Therefore, the identification of effective radiosensitizing agents for the treatment of NPC is urgently needed.

Traditional drug discovery typically involves several key steps, such as identifying targets, screening for lead compounds, and optimizing their structures. Once candidate compounds have been selected, they are tested in cell cultures and animals, followed by preclinical and clinical phases I, II, and III, culminating in market approval.^[^ ^52^ ^]^ However, this process is both time‐consuming and expensive. Repurposing existing drugs, whose safety, efficacy, and administration routes have already been established, significantly reduces costs and saves time while increasing the chances of successful outcomes. Between 2012 and 2017, the FDA approved 170 repurposed drugs, 60% of which were used for new indications.^[^ ^53^ ^]^ These figures highlight the importance of drug repurposing in drug applications and clinical treatment.

To identify potential radiosensitizing agents, we screened 2256 approved drugs and identified vinburnine.^[^ ^20^ ^]^ Vinburnine is an alkaloid extracted from the *Catharanthus* species and is used as a cerebrovascular dilator to treat cardiovascular and cerebrovascular diseases;^[^ ^54^ ^]^ it was approved for clinical treatment in Italy in 1977. Clinical results have shown that vinburnine improves various parameters of cerebral function and enhances the therapeutic effects of treatments for cerebrovascular diseases, with beneficial effects on hemorheology, oxygen transport, and local circulation.^[^ ^55^, ^56^, ^57^, ^58^ ^]^ Additionally, vinburnine has regulatory and protective effects on neuronal homeostasis.^[^ ^59^ ^]^ Although previous studies have shown the anti‐tumor activity of vinburnine in tumor cells,^[^ ^60^, ^61^ ^]^ its function in NPC has not yet been explored. Our findings revealed that vinburnine directly killed NPC cells and acted as a radiosensitizer during NPC radiotherapy. As a non‐cancer therapeutic drug, vinburnine has advantages such as low toxicity and high safety,^[^ ^62^, ^63^, ^64^, ^65^ ^]^ making it an ideal candidate for further investigation.

Next, we showed that vinburnine directly associated with the EDAR receptor and activated the EDAR‐mediated NFκB signaling pathway. After vinburnine binds to EDAR, EDAR forms a complex with EDARADD, which subsequently recruits molecules that activate the NFκB pathway, including TAB2, TAK1, and TRAF6, ultimately leading to NFκB activation.^[^ ^16^, ^27^, ^28^, ^29^ ^]^ Although EDAR may activate the JNK pathway and promote apoptosis, this effect remains controversial. Compared to other TNF‐related signaling cascades, JNK activation via EDAR is relatively weak, suggesting that the physiological significance of this pathway is far less than that of NF‐κB signaling in EDAR‐mediated responses.^[^ ^16^, ^27^, ^28^ ^]^ Additionally, the EDAR–NF‐κB pathway works in concert with the Wnt/β‐catenin pathway during ectodermal development. The EDAR–NF‐κB axis regulates the expression of Wnt ligands (e.g., Wnt10a/b) and transcription factors such as Lef1, forming a positive feedback loop with Wnt/β‐catenin signaling to drive the morphogenesis of hair follicles and teeth.^[^ ^66^ ^]^ EDAR is essential for the development of hair, teeth, and other ectodermal derivatives.^[^ ^67^ ^]^ However, its role in tumors is not well understood. Some studies have suggested that EDAR is highly expressed in tumors and may contribute to tumorigenesis and progression.^[^ ^68^, ^69^ ^]^ However, further research is required to elucidate the underlying mechanisms. A study by Jonathan Vial et al. demonstrated that EDAR restricted melanoma progression in mice.^[^ ^70^ ^]^ Members of the TNF receptor family can help overcome radiotherapy resistance in cervical cancer,^[^ ^71^ ^]^ increase the radiosensitivity of leukemia cells,^[^ ^72^ ^]^ and act as predictors of radiotherapy and chemotherapy efficacy.^[^ ^73^ ^]^ Furthermore, both innate and adaptive immune cells are closely regulated by members of the TNFSF/TNFRSF family, which play crucial roles in coordinating the costimulatory or coinhibitory mechanisms essential for driving the immune response.^[^ ^74^ ^]^ Our study is also the first to demonstrate the role of the EDAR‐NFκB signaling pathway in radiosensitization in NPC and the function of EDAR in enhancing the T cell immune response.

Under the stress of chemotherapy (e.g., cisplatin) or radiotherapy, NF‐κB signaling can activate caspase‐3 and induce apoptosis in tumor cells.^[^ ^26^, ^75^ ^]^ Activated caspase‐3 can trigger not only apoptosis but also pyroptosis in tumor cells,^[^ ^76^ ^]^ positioning caspase‐3 as a crucial molecular switch linking both cell death pathways. In our study, we observed that Vin+IR treatment activated NF‐κB, which in turn triggered caspase‐3‐dependent apoptosis and pyroptosis in tumor cells.

Pyroptosis is characterized by the release of pro‐inflammatory cytokines such as IL‐1β and IL‐18, along with the release of damage‐associated molecular patterns (DAMPs) and pathogen‐associated molecular patterns (PAMPs) from dying cells.^[^ ^77^ ^]^ Within the tumor microenvironment (TME), pyroptotic cells secrete inflammatory cytokines that play critical roles in the initiation and promotion of immune cell infiltration. The release of DAMPs further activates immune cells and promotes their recruitment to the TME, ultimately triggering anti‐tumor immune responses and inhibiting tumor growth and metastasis.^[^ ^77^ ^]^ Cisplatin‐induced activation of GSDME, along with the release of cytokines such as IL‐12, enhances IFN‐γ expression in T cells within the TME, thereby improving the response to anti‐PD‐L1 therapy.^[^ ^78^ ^]^ Thus, endogenous GSDME plays a dual role in suppressing tumor growth and enhancing tumor‐infiltrating lymphocyte (TIL) function.^[^ ^13^ ^]^ Similarly, we confirmed that combining vinburnine with radiation enhanced GSDME‐mediated pyroptosis in NPC cells, which in turn strengthened the antitumor immune response associated with radiotherapy. Inhibiting GSDME‐mediated pyroptosis in NPC cells can result in resistance to docetaxel, contributing to tumor progression.^[^ ^79^ ^]^ Similarly, the inhibition of caspase‐1 and GSDMD‐mediated pyroptosis induces resistance to paclitaxel in NPC both in vitro and in vivo.^[^ ^80^ ^]^ Additionally, GSDME is a prognostic factor for NPC, with low expression predicting radiation resistance.^[^ ^81^ ^]^ Although there have been studies on pyroptosis in NPC, the mechanisms and regulatory factors involved in this process remain unclear. Our findings indicated that vinburnine targeted EDAR, which activated the NFκB signaling pathway. This activation led to mitochondrial damage and induced the production of ROS, which cleaved caspase‐3, subsequently cleaving GSDME. Ultimately, this cascade resulted in pyroptosis of tumor cells.

Radiotherapy can activate the immune system by inducing immunogenic cell death (ICD), which leads to the release of tumor‐specific antigens and subsequently promotes clonal expansion of tumor‐specific T cell subpopulations.^[^ ^82^, ^83^ ^]^ Moreover, radiotherapy upregulates MHC‐I expression in tumor cells, enhances the infiltration of CD8⁺ and CD4⁺ T cells, and improves antigen recognition, thereby increasing the ability of the immune system to detect and eliminate tumor cells.^[^ ^84^ ^]^ Radiotherapy also reshapes the TIME, converting “cold” tumors characterized by immunosuppression into “hot” tumors with heightened immune activation.^[^ ^85^ ^]^ Radiation promotes the release of pro‐inflammatory mediators and chemokines, such as CXCL9, CXCL10, CXCL11, and CXCL16 from tumor and stromal cells, facilitating the recruitment and infiltration of dendritic cells, macrophages, and T lymphocytes, thereby effectively activating the TIME.^[^ ^40^, ^41^ ^]^ In addition, radiotherapy induces TNF production and enhances the TNF‐TNFR axis,^[^ ^86^, ^87^ ^]^ activating downstream signaling pathways such as NF‐κB and leading to transcription of target genes.^[^ ^88^, ^89^, ^90^ ^]^ The presence of TNF also increases the radiosensitivity of tumor cells and improves the tumor‐killing efficacy of radiotherapy.^[^ ^42^, ^43^, ^44^ ^]^ Our study further demonstrated that vinburnine combined with irradiation (Vin+IR) promoted T cell‐attracting chemokine expression (CCL5 and CX3CL1) through activation of the EDAR‐NFκB signaling pathway, thereby enhancing T cell function in the tumor microenvironment and offering a novel strategy for radiosensitization. There is still room for improvement in in vitro and in vivo experiments involving T cells. Before the experiments, we performed MHC class I matching and extracted T cells from a donor with one matched HLA allele. This approach helped reduce the degree of nonspecific T cell activation. However, more comprehensive MHC class I matching, along with MHC class II matching, would provide a more stringent system to better eliminate “bystander” T cell activation. Additionally, our experimental design involved the adoptive transfer of purified T cells; although some CD4⁺ T cells were included, antigen‐presenting cells such as macrophages and DCs were absent. As a result, effective recognition of MHC class II molecules by tumor cells cannot occur, limiting the ability to model fully specific T cell immunity. Therefore, the observed responses primarily reflected partial antigen‐specific recognition by CD8⁺ T cells through MHC class I molecules expressed on tumor cells.^[^ ^91^ ^]^ We plan to address this issue in future studies.

Our study revealed that vinburnine stabilized EDAR, which increased radiotherapy sensitivity and immune responses by stabilizing IR‐induced EDAR expression. Specifically, vinburnine targeted and activated EDAR, which recruited EDARADD and TRAF6 to form a complex that activated the NFκB signaling pathway, promoting the nuclear translocation of p65/p50 and leading to increased transcription of EDAR, thus amplifying the effects of EDAR‐NFκB signaling. Moreover, the nuclear translocation of p65/p50 enhanced the transcription of CCL5 and CX3CL1, prompting tumor cells to release more T cell chemokines. This promoted the recruitment of T cells and enhanced their ability to recognize and kill tumor cells. Moreover, the combination of vinburnine and radiation exacerbated mitochondrial damage in tumor cells, increasing ROS levels and oxidative stress, and triggering apoptosis and pyroptosis. These results demonstrated that vinburnine had a strong synergistic effect with radiotherapy, killing tumor cells while enhancing antitumor immunity and reducing the required radiation dose, thereby minimizing adverse effects during radiation treatment and achieving better therapeutic outcomes. However, there are still areas for improvement. For example, studies are needed to determine whether the increase in GSDME caused by vinburnine in combination with radiation in nasopharyngeal carcinoma is the main pathway for cytokine release and recruitment of GZMB^+^CD8^+^ T cells. In addition, it remains unclear whether vinburnine is equally effective in other cancers that are primarily treated with chemoradiotherapy.

## 4 Conclusion

Overall, our study revealed a highly safe radiotherapy sensitizer that can be rapidly applied in clinical practice. Compared with the commonly used chemoradiotherapy regimen for NPC (cisplatin combined with radiotherapy), vinburnine had significantly fewer side effects and enhanced the T cell immune response more effectively. Additionally, we elucidated the specific molecular mechanisms by which vinburnine sensitized tumors to radiotherapy. Our findings indicated that vinburnine improved the T cell immune response and the efficacy of radiotherapy by promoting the IR‐induced EDAR‐NFκB‐pyroptosis/cytokine positive feedback signaling pathway. This provides a new and safe option for enhancing radiotherapy for the treatment of NPC.

## 5 Experimental Section

### Cells Culture

All NPC cell lines (HK1, SUNE1, HNE1) and normal nasopharyngeal epithelial cell NP69 were obtained from the Cell Center of Central South University. HEK293T cells were of Clontech origin (Mountain View, CA, USA). The SUNE1, HK1, and NP69 cells were cultured in RPMI‐1640 medium (Gibco, USA) supplemented with 15% fetal bovine serum (FBS, Gibco); the HNE1 and 293T cells were cultured in high glucose DMEM (Gibco, USA), also supplemented with 15% FBS. All cells were incubated with 5% CO_2_ at 37 °C.

### Lentiviral Infection

EDAR knockdown lentiviral plasmids (GeneChem, China) were packaged with PSPAX2 and PMD2G in 293T cells. Cell media were collected, and precipitation was removed by centrifugation. After infecting NPC cells with lentiviral fluid for 48h, the uninfected cells were removed with puromycin.

### Cell Viability Assay

The cells were resuspended after the digestion, and then 2000 cells per well were planted in 96‐well plates and incubated overnight to maintain the cell adhesion. According to the experimental design, the absorbance at 450 nm was measured using the CCK8 reagent following drug and radiation treatment. The CCK8 (Selleck, USA) and medium were combined with a ratio of 1:10, which was added to the 110 µL per well, incubated for 1.5 h, and detected at 450 nm optical density. Based on OD values measured at 48 h, survival rates of cells under different drug concentrations were calculated. Data were processed using GraphPad Prism 9.5. Nonlinear regression (curve fitting) was performed to determine the IC50 values of the vinburnine in NPC cells. Furthermore, based on the OD value at 48 h, the inhibition rates of the vinburnine and radiotherapy on the cells were calculated, and the combined radio‐drug index (CI) was determined using CompuSyn software.

### Clone Formation Assay

According to the experimental design, the treated cells were digested and resuspended, and then implanted into the 6‐well plate at a density of 800 cells per well. Cells were incubated in incubators until visible cell clones appeared. The culture medium was removed, and the well was cleaned twice with PBS, fixed with paraformaldehyde for 20 min, then washed twice with PBS, dyed with crystal violet for 15min, and cleaned the dye. After drying the moisture, took pictures and used Image J software for quantitative analysis.

### Flow Cytometry

Cells were collected after drug and radiation treatment, and samples were prepared according to the kit instructions. Cell apoptosis was detected using Annexin V and PI staining (Beyotime, China). The reactive oxygen species (ROS) levels were measured with the DCFH‐DA probe (Solarbio, China). Once the DCFH‐DA probe has penetrated the cell membrane, it is hydrolyzed into DCFH by esterase. ROS then oxidize it to produce the fluorescent substance DCF, a fluorescent compound, whose intensity correlates with ROS levels.

Mitochondrial membrane potential was evaluated using the JC‐1 assay kit (Beyotime, China). The Mitochondrial Membrane Potential Assay Kit (JC‐1) is a device that utilizes JC‐1 as a fluorescent probe to detect the mitochondrial membrane potential in cells, tissues, or purified mitochondria. When mitochondrial membrane potential is high, JC‐1 forms red fluorescent J‐aggregates in the mitochondrial matrix. When the potential is low, JC‐1 remains in its monomeric form, emitting green fluorescence. Flow cytometry data were analyzed with the FlowJo_V10 software.

### Electron Microscopic Detection of Mitochondrial Damage

After drug and radiation treatment, prepare the cell sample, remove the culture medium, add PBS to clean, and gently scrape off the cells. The cell fluid and precipitation were collected by centrifugation. A 2.5% glutaraldehyde electron microscope fixative (Abiowell, China) was added, and the sample was fixed at room temperature for 30 min in the absence of light, followed by a 2‐h fixation with 1% osmium tetroxide (Abiowell, China). Then, it was dehydrated step by step with acetone (Xilong Scientific Co., Ltd., China). The dehydrating agent and Epon‐812 embedding agent were configured according to the ratios of 3:1, 1:1, and 1:3, respectively, and infiltrated into the sample successively. Overnight embedding with Epon‐812 pure embedding agent. An ultra‐thin microtome (LEICA, Germany) made a 60–90nm ultra‐thin section. It was stained with uranium acetate (Beijing Mirror Technology Co., LTD, China) for 15min, followed by lead citrate (Beijing Mirror Technology Co., LTD, China) for 2min at room temperature. Image acquisition was carried out by a JEM‐1400FLASH transmission electron microscope (JEOL, Japan).

### Animal Experiments

The Ethics Committee of Central South University approved the animal experiments (ethics code: CSU‐2022‐0572). A nasopharyngeal carcinoma tumor‐bearing mouse model was constructed with 6‐week‐old female nude mice (Hunan SJA Laboratory Animal Co., Ltd, China). 1×10^^6^ SUNE1 cells were suspended with 100 µL 1640 medium and injected into the right lower limb of mice. 14 days later, when the tumor volume reached 50mm^3^, vinburnine (MCE, USA, 10mg kg^−1^ day^−1^, 10 days) and radiation (X‐rays, 6mV, 2Gy/day, 5 days) treatments were given respectively. The mice's tumor volume and body weight were recorded every two days. Following treatment, the mice were sacrificed, and the difference in tumor volume between the groups was measured.

### RNA sequencing (RNA‐SEQ)

Prepare SUNE1 cells and divide them into four groups. The control group received no treatment, the Vin group was treated with 5µm vinburnine, the IR group was irradiated with 2Gy rays, and the Vin+IR group was treated with both 5µm vinburnine and 2Gy rays. Cells were collected 48h later. The prepared cell samples were submitted to BGI Genomics Co., Ltd. for RNA sequencing. The methods for data analysis are referenced from published articles.^[^ ^92^ ^]^

### Quantitative Real‐time Polymerase Chain Reaction‌ (qRT‐PCR)

Samples were prepared according to the experimental requirements, RNA was extracted using published experimental methods, and qRT‐PCR was conducted.^[^ ^93^ ^]^ Table S1 (Supporting Information) shows the primers and reagents involved in the experiment.

### Western Blotting

The cells were prepared according to the experimental design. Protein extraction, quantification, electrophoresis, membrane transfer, and antibody incubation were conducted following the published experimental protocol.^[^ ^93^ ^]^ Cell membrane proteins were extracted using the Membrane and Cytosol Protein Extraction Kit (Beyotime, China): Collected cell samples were mixed with reagent A and placed in an ice bath for 10 min. The samples underwent two cycles of freezing in liquid nitrogen and thawing at room temperature. Following this, the supernatant was transferred after centrifugation. Next, reagent B was added, the precipitate was resuspended by vortexing, and the tube was returned to the ice bath for another 10 min. Subsequently, the supernatant was collected by centrifugation as the cell membrane protein solution. Band analysis was conducted using ImageJ software, and the gray values were statistically analyzed. The gray values of the control group were utilized for normalization, allowing for the comparison of protein expression levels among the various groups. The antibodies used in the experiment are shown in Table S2 (Supporting Information).

### Chromatin ImmunoPrecipitation (ChIP Assay)

Tumor cells were collected 48h after treatment with the drug and radiation. The sample was prepared according to the published experimental procedure, and the binding of p65 to the DNA fragment of the associated gene was detected using the ChIP kit (CST, USA).^[^ ^93^ ^]^ The sequence of promoter primers involved in the experiment is shown in Table S1 (Supporting Information).

### Cellular Thermal Shift Assay (CETSA)

CETSA is a method for evaluating drug–protein interactions.^[^ ^94^, ^95^, ^96^ ^]^ When drugs bind to target proteins, the protein conformation becomes more stable, and the rate of denaturation decreases at high temperatures. The binding efficiency can be determined by detecting the residual amount of proteins at various temperatures. To assess binding efficiency, SUNE1 cells were treated with 10µm vinburnine/DMSO for 12h, then collected and divided into six aliquots, each heated to a different temperature for 10min. Protein levels were analyzed by Western blot to assess EDAR stability under thermal conditions.

### Surface‐Plasmon Resonance Assay (SPR)

The EDAR protein sample and vinburnine were submitted to MedChemExpress Co., Ltd (MCE, China) for drug target detection. The CM5 chip was placed in the Biacore T200 (GE Healthcare) instrument. The chip channels were activated using 1‐ethyl‐3‐(3‐dimethylaminopropyl) carbodiimide (EDC, GE Healthcare) and N‐hydroxysuccinimide (NHS, GE Healthcare). The EDAR protein was then fixed onto the four channels of the chip. The drug was diluted to various concentrations in a 96‐well plate and subsequently conjugated to the EDAR protein on the chip, starting with the lowest concentration and increasing incrementally. After each concentration, the chip was regenerated using a 10mm glycine hydrochloride solution. This cycle was repeated until all the substances to be measured have been processed. Sample data were collected using the Biacore T200 Control software (v.2.0, GE Healthcare), with reference channel data subtracted. The data were globally fitted to the 1:1 Langmuir binding model using the Biacore T200 evaluation software (v.2.0, GE Healthcare), yielding binding and dissociation constants.

### Co‐Immunoprecipitation‌ (Co‐IP)

The cells were treated with drugs and radiation for 48 h. Co‐immunoprecipitation was performed according to the instructions for the Protein A+G Agarose kit (Beyotime, China). Subsequently, western blotting confirmed that EDAR (Proteintech, China) formed protein complexes with EDARADD (Abclone, China) and TRAF6 (Immunoway, USA).

### Human T Cells Extraction and Culture

20 mL of peripheral blood was extracted, and T cells were isolated following the instructions provided with the Dynabeads Untouched Human T Cells Kit (Thermo Fisher Scientific, USA). The T cells were subsequently cultured in 1640 medium supplemented with 20% serum. The blood samples have been approved by the Medical Ethics Committee of Xiangya Hospital, Central South University (ethics code: 202310059), and written informed consent was obtained from all participating individuals.

### T Cells Killing Assay

Tumor cells, treated with drug and radiation, were planted in 96‐well plates at a density of 5000 cells per well. The cells were cultured overnight in an incubator, and after the tumor cells had attached to the walls, 10 000 T cells were added to each well. The culture was then continued for an additional 48h. Subsequently, the medium was removed, and the wells were cleaned with PBS three times to remove the suspended T cells. Finally, the viability of tumor cells was detected using the CCK8 reagent.

### T Cells Chemotaxis Assay

After the tumor cells underwent drug and radiotherapy for 48h, the culture supernatants were collected, and cell numbers were counted. Based on the counting results, based on the cell count, an equal volume of supernatant from each group was added to the lower chamber of a Transwell insert. Then,1×10^4 T cells were added to the upper chamber and incubated for another 48h. Finally, fluid from the lower chamber was collected, and the number of migrated T cells was quantified.

### T Cell Function Index Detection

The supernatant from cells treated with the drug and radiation was collected and subsequently added to T cells, with the volume corresponding to the number of cells in each group, followed by a 48‐h culture period. The T cells were then collected through centrifugation. Samples were prepared according to published articles,^[^ ^93^ ^]^ and the T cell function was indexed using flow cytometry. The flow cytometry antibodies used in the experiment are listed in Table S3 (Supporting Information).

### Adoptive Transfer Mouse Model

6‐week‐old female NCG mice from GemPharmatech LLC (China) were housed in standard pathogen‐free conditions. 1×10^6^ SUNE1 cells were injected into the right lower limb of NCG mice. Two weeks later, when the tumor volume reached 100mm^3^, vinburnine (10mg kg^−1^ day^−1^, 10 days, IG), cisplatin (4mg kg^−1^ time^−1^, 3 times, IP), and radiotherapy (X‐rays, 6mV, 4Gy) were administered according to the experimental design. After radiation, 1×10^7^ human T cells were injected through the tail vein.^[^ ^97^, ^98^, ^99^, ^100^ ^]^ The mice's tumor volume and body weight were recorded every two days. When the tumor volume of the control group was greater than 1,500mm^3^, the mice were sacrificed, and the tumors were stripped to prepare flow samples to detect T cell indexes. All experiments were conducted in accordance with the Animal Welfare Law and other relevant regulations on animals and experiments, and were approved by the Experimental Animal Welfare Ethics Review Committee of Central South University (CSU‐2024‐0110).

### Multiplex Immunofluorescence Staining (mIF)

The tissue array was obtained from Shanghai OUTDO BIOTECH CO., LTD., and written informed consent was obtained from all participating individuals. All patients were pathologically diagnosed with nasopharyngeal carcinoma, and tumor stages were determined according to the TNM staging system. Detailed information is presented in Table S4 (Supporting Information). Following the kit instructions, mIF was performed using a Multiple fluorescent staining kit (AFIHC025, AiFang biology, China).

Tissue microarrays were dewaxed by immersion in xylene for 45min, followed by sequential hydration in 100%, 100%, 95%, and 75% ethanol for 5min each. After rinsing, antigen retrieval was performed using a high‐temperature microwave method. Endogenous peroxidase activity was blocked with 3% hydrogen peroxide for 10min. The slides were then blocked with goat serum and incubated overnight at 4 °C with the primary antibody EDAR (1:1000, Proteintech) in a light‐protected, humidified chamber. The corresponding Poly‐HRP secondary antibody was added dropwise and incubated in the dark at room temperature for 50min. TSA fluorescent dye was added and incubated for 10 min. Subsequently, the slides were placed in a 100 °C water bath with antigen retrieval solution for 40 min to elute the antibody. Afterward, another 10‐min peroxidase block and a 15‐min serum block was performed. CD8 antibody (1:1000, Aifang Biological) was then applied and incubated overnight at 4 °C. The secondary antibody and TSA dye steps were repeated. Finally, DAPI was added, and a coverslip was mounted. Images were captured using an inverted fluorescence microscope, and fluorescence intensity was analyzed using Slide Converter software (3DHISTECH). The fluorescence intensity of EDAR expression at each site was statistically analyzed and ranked.^[^ ^101^ ^]^ The top 25% were defined as high EDAR expression, while the bottom 25% were considered low expression. The correlation between EDAR expression and prognosis was then analyzed.

### Statistical Analysis

Data are presented as mean ± SD deviation for all results from at least three repeated experiments. The unpaired Student's T‐test was used to compare differences between two groups, while one‐way ANOVA or two‐way ANOVA tests were utilized to compare differences among multiple groups. The Mann‐Whitney test was used to compare gene expression between groups. Pearson correlation analysis and simple linear regression were applied to assess correlations between factors. Survival data were analyzed using Kaplan‐Meier survival analysis. GraphPad Prism 9.5 was used for statistical analyses. Statistical significance was defined as p < 0.05. Levels of significance were indicated as follows: ^*^ *p* < 0.05, ^**^ *p* < 0.01, ^***^ *p* < 0.001, ^****^ *p* < 0.0001, and ns for non‐significant.

### Ethics Statements

All animal experiments were approved by the Experimental Animal Welfare Ethics Review Committee of Central South University (approval numbers: CSU‐2022‐0572 and CSU‐2024‐0110) and conducted in accordance with institutional guidelines. The study protocol involving human participants was reviewed and approved by the Medical Ethics Committee of Xiangya Hospital, Central South University (approval number: 202310059). All participants were informed of the details of the study, and signed informed consent.

## Conflict of Interest

The authors declare no conflict of interest.

## Author Contributions

J.C. and N.L. contributed equally to this work. J.C. and N.L. carried out experiments. J.C. and Q.T. performed statistical analysis. J.W. and J.H. analyzed clinic data. C.P., X.C., and L.S supervised the study. C.P. and N.L. conceptualized and wrote the manuscript. All authors approved the final manuscript. C.P. is the guarantor of this work, who takes responsibility for the integrity of the entire study and the accuracy of the data.

## Supporting information



Supporting Information

## Data Availability

All the data during the current study are available within the paper and its Supplementary information, or from the corresponding author upon reasonable request.
