# Supplementary material for: Vinburnine Sensitizes Radiotherapy Efficacy in Nasopharyngeal Carcinoma by Triggering Pyroptosis and Immune Responses via Activation of EDAR‐NFκB Pathway
Source: Adv Sci (Weinh). 2025 Sep 25;12(46):e06139. doi: 10.1002/advs.202506139 (PMC12697895; doi:10.1002/advs.202506139)
Supplement: Supplementary file 1 — Supporting Information [file ADVS-12-e06139-s001.docx]

Supplementary Materials for

**Vinburnine Sensitizes Radiotherapy Efficacy in Nasopharyngeal Carcinoma by Triggering Pyroptosis and Immune Responses via Activation of EDAR-NFκB Pathway**

Jing Chen *et al.*

*Corresponding author. Email: pengcongxy@csu.edu.cn

**This file includes:**

Supplementary Text

Figures. S1 to S7

Tables S1 to S5

**Supplementary Figures:**

**
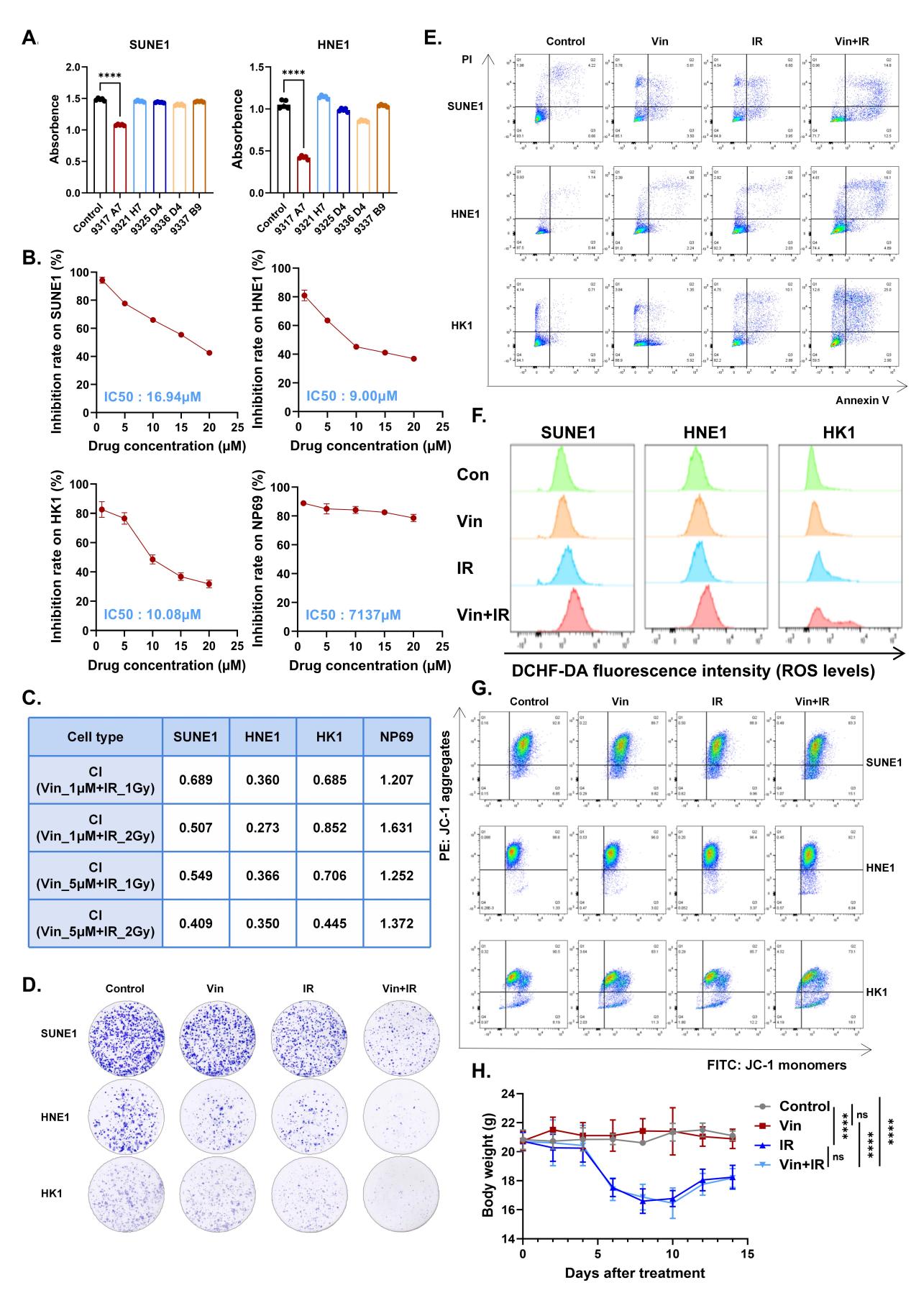
**

**Figure S1.** Vinburnine sensitizes radiotherapy efficacy in NPC cells. A) CCK8 was used to detect the cell viability of SUNE1 and HNE1 cells after 6 FDA-medicines treatment (10μM) for 48h (n=5). B) The IC50 values of vinburnine in NPC cells and NP69 cells were calculated based on the OD values at 48h. C) The CI index of Vin and IR was analyzed and calculated using the CompuSyn software. D) NPC cells clone formation was detected after 5μM Vin and 2Gy IR treatment for 48h. E-G) Flow cytometry was used to detect apoptosis/ROS levels/mitochondrial membrane potential of treated cells (n=3). H) Weight curves of SUNE1-bearing mice with the indicated treatments (n=7). Multiple samples were presented using mean ± standard deviation (SD). A), H) Statistical analysis with One-way ANOVA was used to analyze the statistical differences among multiple groups. *p < 0.05, **p < 0.01, ***p < 0.001, ****p < 0.0001, ns for non-significant.

**
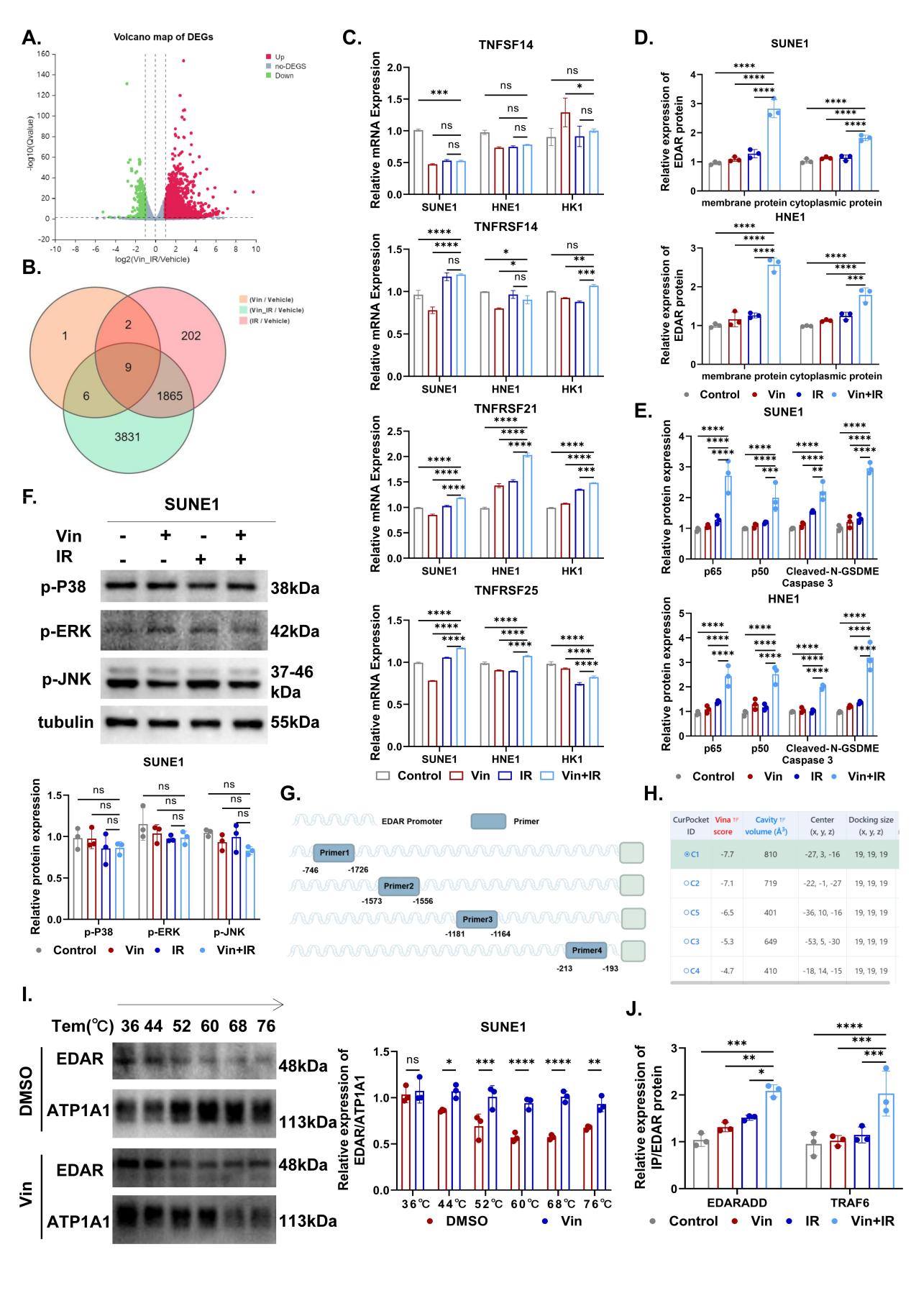
**

**Figure S2.** Validation of Vinburnine's target and pathway. A) Volcano map of differential genes expression in transcriptomics. B) The number of intersections of sample genes in different classes of transcriptome. C) Detection of mRNA levels of transcriptomic differential genes in treated cells (n=2). D) The expression level of the EDAR protein was quantitatively analyzed by using ImageJ software (n=3). E) The expression level of the p65/p50/Cleaved-caspase 3/N-GSDME were quantitatively analyzed by using ImageJ software (n=3). F) Western blotting was used to detect the MAPK signaling pathway proteins (p-P38/p-ERK/p-JNK) (upper panel). The expression level of the p-P38/p-ERK/p-JNK were quantitatively analyzed by using ImageJ software (lower panel) (n=3). G) Primers designed based on the p65 binding EDAR promoter site predicted by the JASPAR database. H) The CB-Dock2 website predicts that there are a total of 5 binding sites for vinburnine and EDAR proteins, among which the one with the largest binding fraction is -7.7. I) CESTA experiment validates Vinburnine in combination with EDAR. Cells were collected following treatment with 10μM Vin/DMSO for 12h, and proteins were extracted after exposure to various temperatures for 10min. The expression of EDAR was detected by Western blotting (left panel). Statistical analysis of EDAR expression levels in each group (right panel) (n=3). J) Statistical analysis of the relative expression levels of EDARADD and TRAF6 proteins compared to EDAR in each group of IP samples for the CoIP experiment (n=3). Multiple samples were presented using mean ± standard deviation (SD). C-E), I-J) Statistical analysis with Two-way ANOVA was used to analyze the statistical differences among multiple groups. *p < 0.05, **p < 0.01, ***p < 0.001, ****p < 0.0001, ns for non-significant.

**
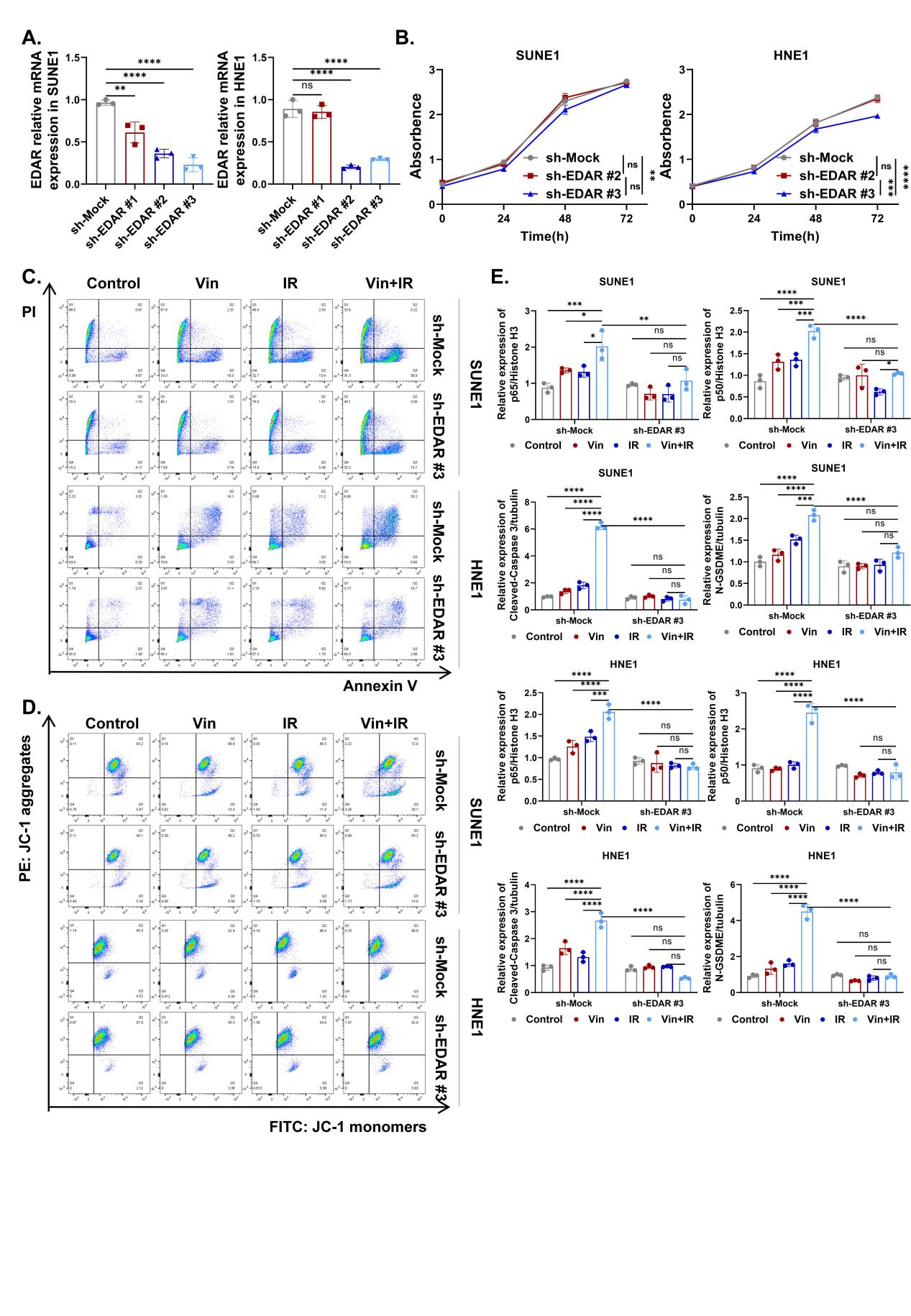
**

**Figure S3.** Knockdown of EDAR suppresses apoptosis and mitochondrial membrane potential. A) The relative mRNA expression of EDAR in sh-Mock or sh-EDAR NPC cells was determined (n=3). B) CCK8 was used to detect the proliferation of NPC cells after sh-EDAR (n=5). C-D) Flow cytometry was used to detect the apoptosis/mitochondrial membrane potential of the treated cells after EDAR knockdown (n=3). E) The western blotting bands of p65, p50, cleaved-caspase3, and N-GSDME were analyzed for grayscale intensity and quantified using ImageJ software. Multiple samples were presented using mean ± standard deviation (SD). A-B) Statistical analysis with One-way ANOVA was used to analyze the statistical differences among multiple groups. E) Statistical analysis with Two-way ANOVA was used to analyze the p65/p50/cleaved-caspase3/N-GSDME expression levels among multiple groups. *p < 0.05, **p < 0.01, ***p < 0.001, ****p < 0.0001, ns for non-significant.

**
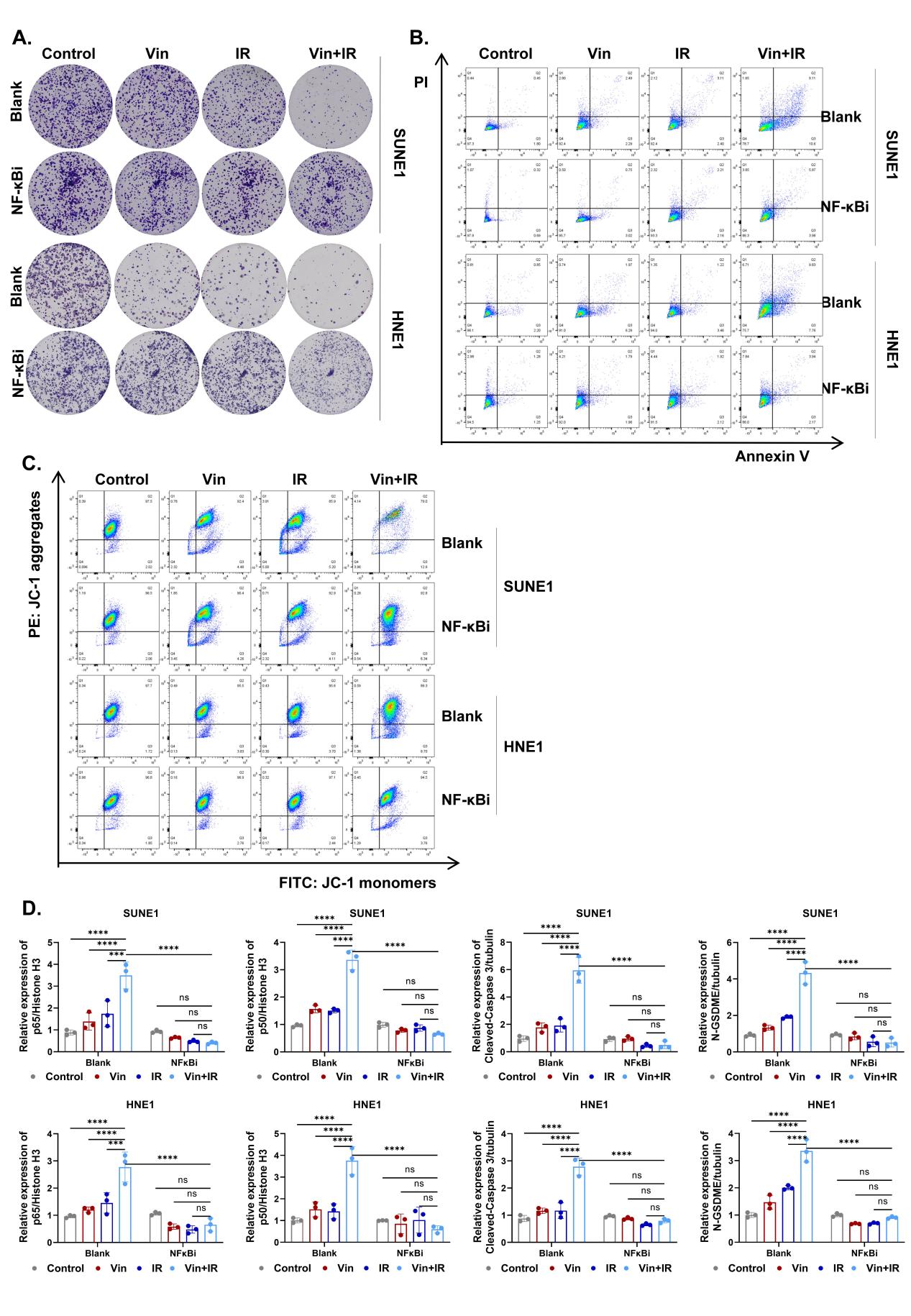
**

**Figure S4.** NFκB inhibitor suppresses the radiosensitizing effect of vinburnine. A) After pretreatment with the NFκBi for 24h, the cell clone formation ability of the 5μM Vin and 2Gy IR was detected (n=3). B-C) Flow cytometry detected the apoptosis and mitochondrial membrane potential of the Vin±IR-treated cells following NFκBi pretreatment (n=3). D) The western blotting bands of p65, p50, cleaved-caspase3, and N-GSDME were analyzed for grayscale intensity and quantified using ImageJ software, and statistical analysis with Two-way ANOVA was used to analyze the p65/p50/cleaved-caspase3/N-GSDME expression levels among multiple groups (n=3). *p < 0.05, **p < 0.01, ***p < 0.001, ****p < 0.0001, ns for non-significant.

**
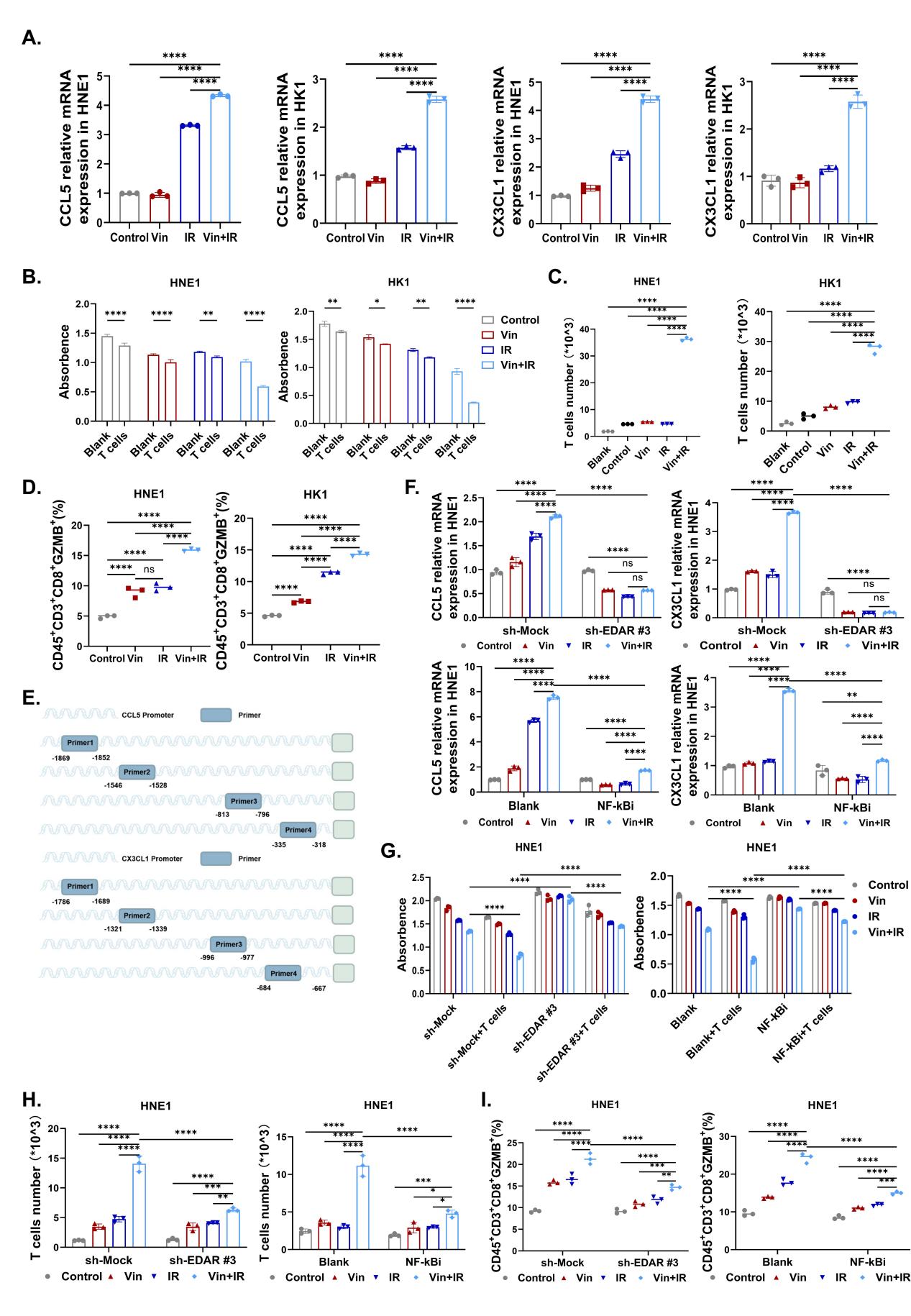
**

**Figure S5.** Vin+IR promotes the expression of CCL5/CX3CL1 through the EDAR-NFκB pathway, and enhances the recruitment and cytotoxicity of T cells. A) The mRNA expression of CCL5 and CX3CL1 in HNE1 and HK1 after 5μM Vin/ 2Gy IR treatment (n=3). B) After 24h of 5μM Vin/ 2Gy IR treatment, HNE1 (n=5) or HK1 (n=3) were co-cultured with T cells for 48h, and cell viability was detected by CCK8. C) The number of chemotactic T cells in the treated HNE1 or HK1 cell supernatant (n=3). D) T cell ratio of CD45^+^CD3^+^CD8^+^GZMB^+^ after being treated with HNE1 or HK1 cell supernatant for 48h (n=3). E) Primers designed based on the p65 binding CCL5 and CX3CL1 promoter site predicted by the JASPAR database. F) After sh-EDAR or NFκBi pretreatment, the mRNA expression of CCL5 and CX3CL1 in Vin/IR-treated HNE1 or HK1 was detected using qRT-PCR (n=3). G-I) Following sh-EDAR or NFκBi pretreatment, the proliferation of HNE1 cells treated with 5μM Vin/ 2Gy IR and co-cultured with T cells was measured, as well as the number of chemotactic T cells and the proportion of CD45^+^CD3^+^CD8^+^GZMB^+^ T cells in the supernatant of Vin/IR-treated HNE1 cells (n=3). Multiple samples were presented using mean ± standard deviation (SD). A), C-D) Statistical analysis with One-way ANOVA was used to analyze the statistical differences among multiple groups. B), F-I) Statistical analysis with Two-way ANOVA was used to analyze the statistical differences among multiple groups. *p < 0.05, **p < 0.01, ***p < 0.001, ****p < 0.0001, ns for non-significant.


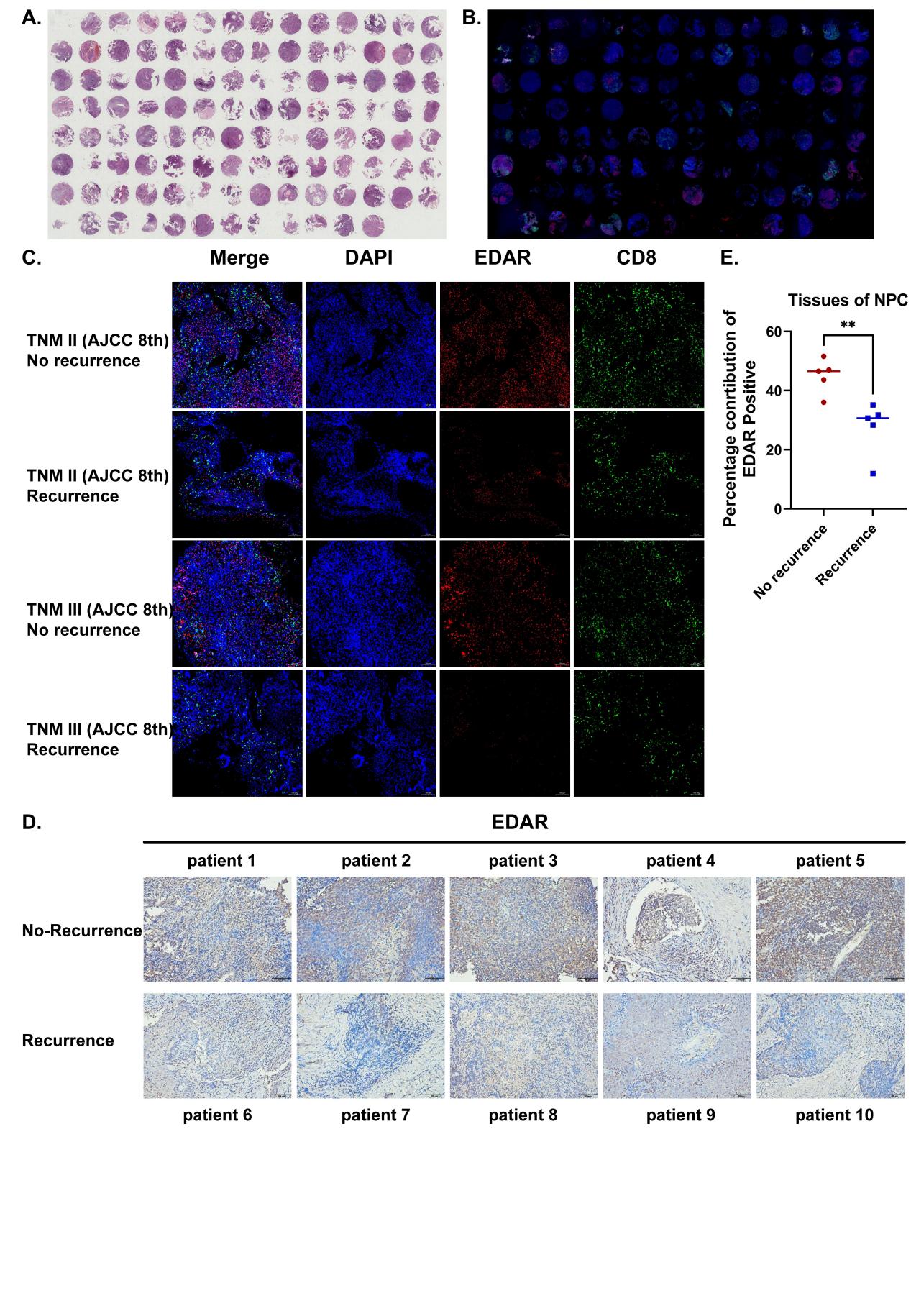


**Figure S6.** The expression of EDAR and CD8 in NPC tissue array. A) The HE staining images of nasopharyngeal carcinoma tissue chips were provided by Shanghai OUTDO BIOTECH CO., LTD (Scale bar, 2000μm). B) Multiple immunofluorescence staining images of nasopharyngeal cancer tissue (Scale bar, 2000μm). Note: The tissue microarray comprises a total of 110 site tissues. Following multiple staining and fluorescence analysis, 26 sites exhibited delamination or an insufficient effective area (A01, A08, A14, B09, B10, C02, C04, C09, D06, D11, D13, E01, E09, F03, F06, F07, F12, F14, G06, G07, G09, H01, H04, H08, H09, H10). Consequently, a total of 84 sites were subjected to fluorescence subanalysis, with 39 being from patients without recurrence and 45 from patients with recurrence. C) Representative images of EDAR and CD8 in recurrent (n=39) and non-recurrent (n=45) NPC tissue (Scale bar, 100μm). D) Pathological specimens of NPC with recurrence (n=5) and those without recurrence (n=5) within 5 years after receiving the standard treatment regimen (induction chemotherapy followed by concurrent chemoradiotherapy) were selected for EDAR IHC staining (Scale bar, 200μm). E) The positive expression rate was analyzed using the ImageJ IHC profiler, and statistical analysis with a T-test was conducted on the two groups using GraphPad Prism 9.5 software. *p < 0.05, **p < 0.01, ***p < 0.001, ****p < 0.0001, ns for non-significant.


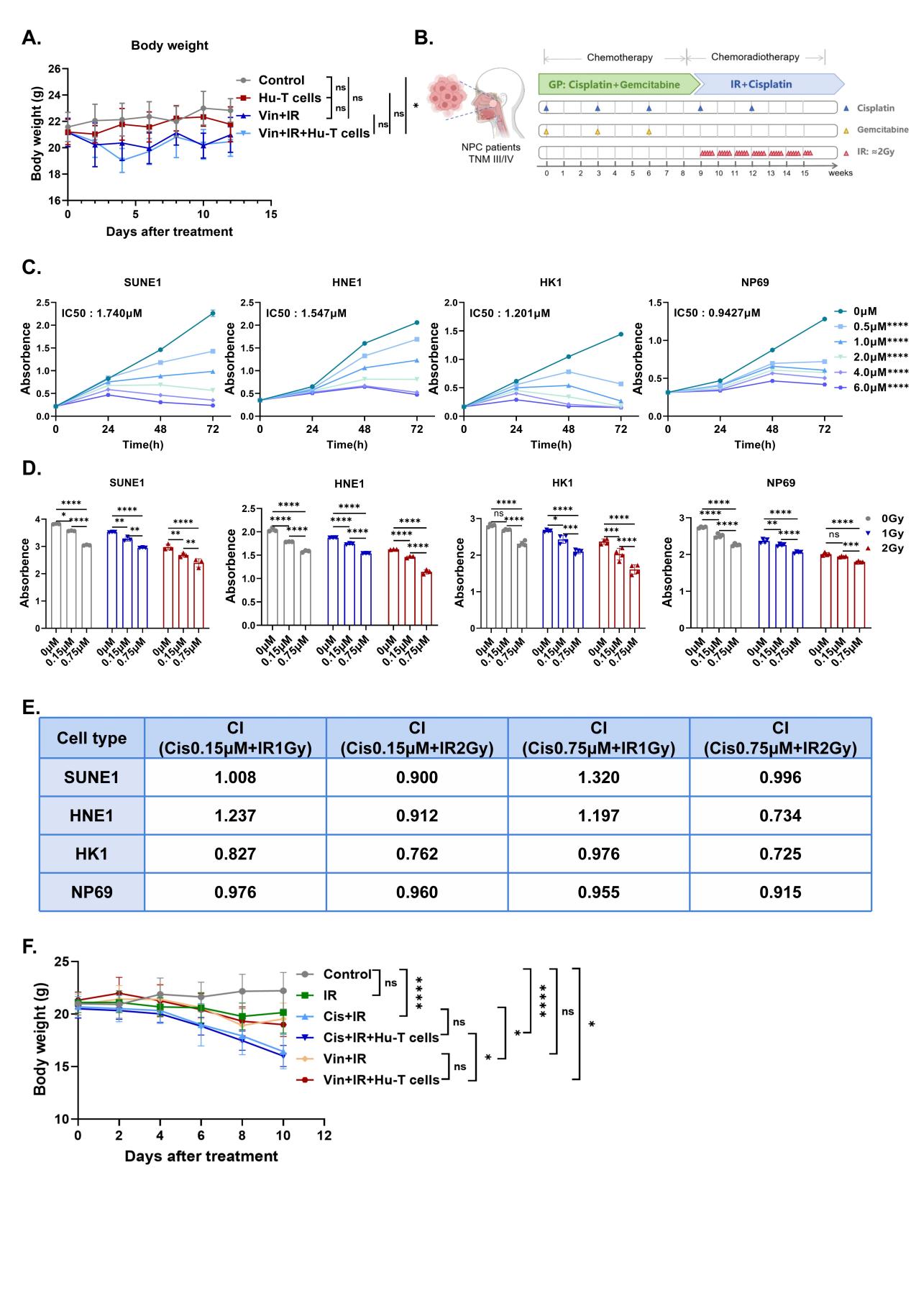


**Figure S7.** The radiosensitizing effect of cisplatin on NPC and normal nasopharyngeal epithelial cells. A) Weight curves of SUNE1-bearing mice with the indicated treatments (n=5). B) Schematic diagram of the clinical treatment plan for nasopharyngeal carcinoma. C) CCK8 was used to detect the proliferation of NPC cells and NP69 after (0-6μM) Cisplatin treatment for 0-72h (n=4). D) The effects of Cis combined with radiation on NPC cells (SUNE1/HNE1 n=3, HK1 n=4) and NP69 (n=4) were detected by CCK8 for 48h. E) The CI index of Cis and IR was analyzed and calculated by the CompuSyn software. F) Weight curves of SUNE1-bearing mice with the indicated treatments (n=6). Multiple samples were presented using mean ± standard deviation (SD). A), C), F) Statistical analysis with One-way ANOVA was used to analyze the statistical differences among multiple groups. D) Statistical analysis with Two-way ANOVA was used to analyze the statistical differences among multiple groups. *p < 0.05, **p < 0.01, ***p < 0.001, ****p < 0.0001, ns for non-significant.

**Supplementary Table:**

**Table S1. The primers used in the PCR reaction and annealing.**

| **REAGENT or RESOURCE** |  |
| --- | --- |
| Gene name | Sequence (5'to3') |
| EDAR primer 1 | F: GCCTATAAGATTGCCACATAG  R: TTGAGAAGCACTGGTCTAG |
| EDAR primer 2 | F: AGGTGATGAGGTCGTGAG  R: GGACTGGCTAATGAACTGAA |
| EDAR primer 3 | F: GGCACTGATGGAGATTGG  R: CAGATACAGGCAGGAGGT |
| EDAR primer 4 | F: CCTCTCAGAGAATTGGTAACA  R: CCGCACCTTAGTGTCAAC |
| CCL5 primer 1 | F: GTCCTAACTGCCACTCCT  R: TTCTCTGCTGACATCCTTAG |
| CCL5 primer 2 | F: GTGGCTGGCACAGATAAG  R: GTTGGAATGAGTCCTCTTGT |
| CCL5 primer 3 | F: AGACCATCCTGGCTAACAT  R: GCACAATCTCGGCTCATT |
| CCL5 primer 4 | F: GTTCACGCCATTCTCCTG  R: GGTTCACGCCTGTAATCC |
| CX3CL1 primer 1 | F: GGTGTTCCGCTACTTCAA  R: CTGCCTTGGTATAGGACTG |
| CX3CL1 primer 2 | F: GCAGACTGTGTTCTAATGTG  R: TCTTGGCTCCTCAACTCT |
| CX3CL1 primer 3 | F: GTCCACATTCTGCTCATCA  R: GGTCTGACAAGCTCTTCTT |
| CX3CL1 primer 4 | F: TGTGTGTTGCCCACTTAG  R: CAGGATGTTGCCAAGGAA |
| ACTB (Human) | F: ATCGGCGGCTCCATCCTG  R: GACTCGTCATACTCCTGCTTGC |
| EDAR (Human) | F: CTGAGAAGGCTGTTGTGAAAAC  R: ATCTGCACCAGTTTTGTGAGTA |
| CCL5 (Human) | F: CCTCGCTGTCATCCTCATTGC  R: ACTTGCCACTGGTGTAGAAATACTC |
| CX3CL1 (Human) | F: TCGTGGCTGCTCCGCTTG  R: CCTGGTTCTGTTGATAGTGGATGAG |
| TNFSF14 (Human) | F: CTGTTATGGGAGACTCAGCTG  R: ATGTAGTAGTAGCCAGCTTTGG |
| TNFRSF14 (Human) | F: AGTCCAGGTTATCGTGTGAAG  R: AGACACTTGCTTAGGCCATT |
| TNFRSF21 (Human) | F: GCATGTTCCAGTCTAACGCTACC  R: ACACCGCACATCCTCAGTCTC |
| TNFRSF25 (Human) | F: TTCTACTGCCAACCATGCCTAG  R: CATCGCCATGTTCATAGAAGC |

|  |
| --- |

**Table S2. The antibody used in the western blotting.**

| **REAGENT** | **SOURCE** | **IDENTIFIER** |
| --- | --- | --- |
| **Antibody** |  |  |
| EDAR | ProteinTech | Cat No. 18032-1-AP |
| ATP1A1 | ProteinTech | Cat No. 14418-1-AP |
| tubulin | ProteinTech | Cat No. 11224-1-AP |
| NFκB p65 | ProteinTech | Cat No. 10745-1-AP |
| NFκB p50 | ProteinTech | Cat No. 14220-1-AP |
| Histone H3 | ProteinTech | Cat No. 17168-1-AP |
| GAPDH | ProteinTech | Cat No. 10494-1-AP |
| Cleaved-Caspase 3 | ProteinTech | Cat No. 25128-1-AP |
| p-JNK | ProteinTech | Cat No. 80024-1-RR |
| GSDME | Abcam | Cat No. 215191 |
| EDARADD | ABclonal | Cat No. A15950 |
| TRAF6 | Immunoway | Cat No. YT4720 |
| p-P38 | Santa Cruz Biotechnology | Cat No. sc-166182 |
| PI3K | Cell Signaling Technology | Cat No. 4255S |

**Table S3. The antibody used in the T cell function index detection.**

| **REAGENT** | **SOURCE** | **IDENTIFIER** |
| --- | --- | --- |
| **Antibody** |  |  |
| TruStain FcX (anti-mouse CD16/32) Antibody | BioLegend | Cat No. 422301 |
| PerCP/Cyanine5.5 anti-human CD45 | BioLegend | Cat No. 304028 |
| FITC anti-human CD3 | BioLegend | Cat No. 300406 |
| Brilliant Violet 605™ anti-human CD8 | BioLegend | Cat No. 344742 |
| PE anti-human IFN-γ | BioLegend | Cat No. 502509 |
| APC anti-human/mouse Granzyme B Recombinant | BioLegend | Cat No. 396408 |

**Table S4. Microarray information of nasopharyngeal carcinoma tissues.**

| Point | Diagnosis time | Survival status | Follow-up time | OS (M) | T | N | M | AJCC 8th | Recurrence time |
| --- | --- | --- | --- | --- | --- | --- | --- | --- | --- |
| A01 | 2010.01.13 | Survival | 2017.03 | 86 | T2 | N2 | M0 | III | —— |
| A02 | 2010.01.18 | Survival | 2017.03 | 86 | T2 | N0 | M0 | II | —— |
| A03 | 2010.01.18 | Survival | 2017.03 | 86 | T1 | N0 | M0 | I | —— |
| A04 | 2010.02.02 | Survival | 2017.03 | 85 | T2 | N1 | M0 | II | —— |
| A05 | 2010.02.03 | Survival | 2017.03 | 85 | T1 | N0 | M0 | I | —— |
| A06 | 2010.02.06 | Survival | 2017.03 | 85 | T2 | N0 | M0 | II | —— |
| A07 | 2010.02.06 | Survival | 2017.03 | 85 | T3 | N3 | M0 | IVa | 2014-09 |
| A08 | 2010.02.10 | Survival | 2017.03 | 85 | T2 | N1 | M0 | II |  |
| A09 | 2010.02.25 | Death | 2015/10/1 | 68 | T4 | N1 | M0 | IVa | 2011-05 |
| A10 | 2010.03.04 | Survival | 2017.03 | 84 | T3 | N3 | M0 | IVa | 2016-07 |
| A11 | 2010.03.09 | Survival | 2017.03 | 84 | T1 | N0 | M0 | I | —— |
| A12 | 2010.03.12 | Survival | 2017.03 | 84 | T2 | N1 | M0 | II | —— |
| A13 | 2010.03.12 | Death | 2014/5/1 | 50 | T3 | N2 | M0 | III | —— |
| A14 | 2010.03.16 | Survival | 2017.03 | 84 | T1 | N0 | M0 | I | —— |
| B01 | 2010.03.19 | Survival | 2017.03 | 84 | T3 | N1 | M0 | III | —— |
| B02 | 2010.03.24 | Death | 2014/10/1 | 55 | T2 | N0 | M0 | II | 2013-12 |
| B03 | 2010.03.24 | Survival | 2017.03 | 84 | T1 | N0 | M0 | I | —— |
| B04 | 2010.03.29 | Survival | 2017.03 | 84 | T3 | N1 | M0 | III | —— |
| B05 | 2010.04.07 | Death | 2016/11/1 | 79 | T4 | N2 | M0 | IVa | 2014-12 |
| B06 | 2010.04.09 | Survival | 2017.03 | 83 | T2 | N1 | M0 | II | —— |
| B07 | 2010.04.22 | Death | 2014/11/1 | 55 | T4 | N2 | M0 | IVa | 2014-09 |
| B08 | 2010.04.25 | Survival | 2017.03 | 83 | T2 | N2 | M0 | III | —— |
| B09 | 2010.04.27 | Survival | 2017.03 | 83 | T1 | N0 | M0 | I | —— |
| B10 | 2010.04.30 | Survival | 2017.03 | 83 | T2 | N1 | M0 | II | —— |
| B11 | 2010.05.10 | Survival | 2017.03 | 82 | T3 | N1 | M0 | III | —— |
| B12 | 2010.05.15 | Survival | 2017.03 | 82 | T2 | N0 | M0 | II | —— |
| B13 | 2010.05.17 | Death | 2015/5/1 | 60 | T4 | N2 | M0 | IVa | 2014-08 |
| B14 | 2010.05.25 | Death | 2016/11/1 | 78 | T3 | N2 | M0 | III | 2014-11 |
| C01 | 2010.05.24 | Survival | 2017.03 | 82 | T2 | N2 | M0 | III | —— |
| C02 | 2010.06.02 | Survival | 2017.03 | 81 | T2 | N1 | M0 | II | —— |
| C03 | 2010.06.09 | Death | 2014/7/1 | 49 | T4 | N2 | M1 | IVb | 2014-02 |
| C04 | 2010.06.11 | Survival | 2017.03 | 81 | T2 | N0 | M0 | II | —— |
| C05 | 2010.06.13 | Death | 2015/11/1 | 65 | T3 | N3 | M0 | IVa | 2013-03 |
| C06 | 2010.06.13 | Survival | 2017.03 | 81 | T3 | N1 | M0 | III | —— |
| C07 | 2010.06.18 | Survival | 2017.03 | 81 | T2 | N1 | M0 | II | —— |
| C08 | 2010.06.24 | Survival | 2017.03 | 81 | T2 | N0 | M0 | II | 2013-09 |
| C09 | 2010.06.28 | Survival | 2017.03 | 81 | T3 | N1 | M0 | III | —— |
| C10 | 2010.06.24 | Survival | 2017.03 | 81 | T2 | N1 | M0 | II | —— |
| C11 | 2010.06.28 | Death | 2016/8/1 | 74 | T3 | N3 | M0 | IVa | 2012 |
| C12 | 2010.06.28 | Survival | 2017.03 | 81 | T1 | N0 | M0 | I | —— |
| C13 | 2010.06.29 | Survival | 2017.03 | 81 | T2 | N2 | M0 | III | —— |
| C14 | 2010.07.08 | Survival | 2017.03 | 80 | T2 | N2 | M0 | III | 2014-09 |
| D01 | 2010.07.13 | Survival | 2017.03 | 80 | T3 | N2 | M0 | III | —— |
| D02 | 2010.07.16 | Survival | 2017.03 | 80 | T2 | N1 | M0 | II | —— |
| D03 | 2010.07.19 | Survival | 2017.03 | 80 | T3 | N1 | M0 | III | —— |
| D04 | 2010.07.26 | Survival | 2017.03 | 80 | T2 | N1 | M0 | II | —— |
| D05 | 2010.08.02 | Survival | 2017.03 | 79 | T1 | N0 | M0 | I | —— |
| D06 | 2011.01.13 | Death | 2015/7/1 | 54 | T4 | N2 | M0 | IVa | 2014-09 |
| D07 | 2011.01.27 | Survival | 2017.03 | 74 | T2 | N1 | M0 | II | —— |
| D08 | 2011.02.12 | Survival | 2017.03 | 73 | T3 | N1 | M0 | III | —— |
| D09 | 2011.02.12 | Death | 2016/8/1 | 66 | T4 | N2 | M0 | IVa | 2015-03 |
| D10 | 2011.02.18 | Survival | 2017.03 | 73 | T3 | N2 | M0 | III | 2016-02 |
| D11 | 2011.02.20 | Survival | 2017.03 | 73 | T2 | N1 | M0 | II | 2013-05 |
| D12 | 2011.02.22 | Survival | 2017.03 | 73 | T1 | N0 | M0 | I | —— |
| D13 | 2011.02.25 | Survival | 2017.03 | 73 | T2 | N1 | M0 | II | —— |
| D14 | 2011.02.28 | Survival | 2017.03 | 73 | T3 | N2 | M0 | III | 2014-04 |
| E01 | 2011.03.07 | Survival | 2017.03 | 72 | T3 | N1 | M0 | III | —— |
| E02 | 2011.03.08 | Survival | 2017.03 | 72 | T1 | N0 | M0 | I | —— |
| E03 | 2011.03.14 | Survival | 2017.03 | 72 | T3 | N2 | M0 | III | 2015-06 |
| E04 | 2011.03.17 | Survival | 2017.03 | 72 | T2 | N1 | M0 | II | —— |
| E05 | 2011.03.17 | Survival | 2017.03 | 72 | T3 | N2 | M0 | III | 2013-09 |
| E06 | 2011.03.29 | Survival | 2017.03 | 72 | T3 | N2 | M0 | III | 2015-07 |
| E07 | 2011.04.06 | Survival | 2017.03 | 71 | T3 | N1 | M0 | III | —— |
| E08 | 2011.04.21 | Survival | 2017.03 | 71 | T1 | N0 | M0 | I | —— |
| E09 | 2011.04.23 | Death | 2014/12/1 | 32 | T4 | N1 | M0 | IVa | 2012-05 |
| E10 | 2011.04.26 | Survival | 2017.03 | 71 | T2 | N2 | M0 | III | 2014-02 |
| E11 | 2011.05.08 | Survival | 2017.03 | 70 | T2 | N0 | M0 | II | 2015-01 |
| E12 | 2011.05.16 | Survival | 2017.03 | 70 | T3 | N2 | M0 | III | 2016-02 |
| E13 | 2011.05.18 | Survival | 2017.03 | 70 | T2 | N1 | M0 | II | 2014-07 |
| E14 | 2011.05.22 | Death | 2015/11/1 | 54 | T2 | N3 | M0 | IVa | 2014-11 |
| F01 | 2011.05.23 | Survival | 2017.03 | 70 | T2 | N0 | M0 | II | —— |
| F02 | 2011.05.25 | Survival | 2017.03 | 70 | T3 | N2 | M0 | III | 2014-08 |
| F03 | 2011.05.31 | Survival | 2017.03 | 70 | T2 | N1 | M0 | II | 2013-11 |
| F04 | 2011.06.03 | Survival | 2017.03 | 69 | T2 | N0 | M0 | II | 2015-04 |
| F05 | 2011.06.08 | Survival | 2017.03 | 69 | T2 | N1 | M0 | II | —— |
| F06 | 2011.06.08 | Survival | 2017.03 | 69 | T2 | N1 | M0 | II | —— |
| F07 | 2011.06.14 | Survival | 2017.03 | 69 | T2 | N2 | M0 | III | 2013-11 |
| F08 | 2011.06.16 | Survival | 2017.03 | 69 | T2 | N0 | M0 | II | —— |
| F09 | 2011.06.17 | Survival | 2017.03 | 69 | T3 | N2 | M0 | III | 2015-02 |
| F10 | 2011.06.20 | Survival | 2017.03 | 69 | T1 | N0 | M0 | I | —— |
| F11 | 2011.06.20 | Survival | 2017.03 | 69 | T2 | N0 | M0 | II | 2013-07 |
| F12 | 2011.06.28 | Survival | 2017.03 | 69 | T3 | N2 | M0 | III | 2014-06 |
| F13 | 2011.06.29 | Survival | 2017.03 | 69 | T2 | N0 | M0 | II | 2015-03 |
| F14 | 2011.06.29 | Survival | 2017.03 | 69 | T2 | N0 | M0 | II | 2013-12 |
| G01 | 2011.07.10 | Survival | 2017.03 | 68 | T1 | N0 | M0 | I | —— |
| G02 | 2011.07.11 | Survival | 2017.03 | 68 | T2 | N0 | M0 | II | 2015-09 |
| G03 | 2011.07.14 | Survival | 2017.03 | 68 | T2 | N1 | M0 | II | 2013-12 |
| G04 | 2011.07.19 | Survival | 2017.03 | 68 | T2 | N2 | M0 | III | 2014-06 |
| G05 | 2011.07.20 | Survival | 2017.03 | 68 | T2 | N1 | M0 | II | 2014-04 |
| G06 | 2011.07.25 | Survival | 2017.03 | 68 | T2 | N1 | M0 | II | 2013-12 |
| G07 | 2011.08.01 | Death | 2014/6/1 | 34 | T4 | N2 | M0 | IVa | 2013-11 |
| G08 | 2011.08.09 | Survival | 2017.03 | 67 | T2 | N1 | M0 | II | —— |
| G09 | 2011.08.21 | Survival | 2017.03 | 67 | T3 | N1 | M0 | III | —— |
| G10 | 2011.08.23 | Death | 2016/7/1 | 59 | T4 | N1 | M0 | IVa | 2014-03 |
| G11 | 2011.08.24 | Survival | 2017.03 | 67 | T2 | N0 | M0 | II | —— |
| G12 | 2011.08.28 | Survival | 2017.03 | 67 | T1 | N2 | M0 | III | 2015-09 |
| G13 | 2011.08.28 | Survival | 2017.03 | 67 | T2 | N2 | M0 | III | 2014-01 |
| G14 | 2011.08.30 | Death | 2016/11/1 | 63 | T4 | N2 | M0 | IVa | 2014-07 |
| H01 | 2011.09.01 | Survival | 2017.03 | 66 | T1 | N0 | M0 | I | —— |
| H02 | 2011.09.01 | Survival | 2017.03 | 66 | T2 | N1 | M0 | II | —— |
| H03 | 2011.09.05 | Survival | 2017.03 | 66 | T2 | N1 | M0 | II | —— |
| H04 | 2011.09.13 | Death | 2016/12/1 | 63 | T4 | N1 | M0 | IVa | 2015-03 |
| H05 | 2011.09.19 | Survival | 2017.03 | 66 | T2 | N1 | M0 | II | —— |
| H06 | 2011.09.19 | Survival | 2017.03 | 66 | T3 | N0 | M0 | III | 2015-04 |
| H07 | 2011.09.19 | Survival | 2017.03 | 66 | T2 | N1 | M0 | II | —— |
| H08 | 2011.09.21 | Survival | 2017.03 | 66 | T2 | N1 | M0 | II | —— |
| H09 | 2011.09.23 | Survival | 2017.03 | 66 | T3 | N1 | M0 | III | —— |
| H10 | 2011.09.29 | Survival | 2017.03 | 66 | T3 | N1 | M0 | III | —— |
| H11 | 2011.10.08 | Survival | 2017.03 | 65 | T4 | N1 | M0 | IVa | 2014-05 |
| H12 | 2011.10.09 | Survival | 2017.03 | 65 | T2 | N0 | M0 | II | 2015-06 |

Note: The tissue microarray comprises a total of 110 site tissues. Following multiple staining and fluorescence analysis, 26 sites exhibited delamination or an insufficient effective area (A01, A08, A14, B09, B10, C02, C04, C09, D06, D11, D13, E01, E09, F03, F06, F07, F12, F14, G06, G07, G09, H01, H04, H08, H09, H10). Consequently, a total of 84 sites were subjected to fluorescence subanalysis, with 39 being from patients without recurrence and 45 from patients with recurrence.

**Table S5. NPC patient information of EDAR IHC specimens.**

| **Number** | **TNM (AJCC 8th)** | **Date of diagnosis** | **Recurrence time** |
| --- | --- | --- | --- |
| **1** | T4N3M0 IVa | 2018/3/6 | - |
| **2** | T4N1M0 IVa | 2020/1/10 | - |
| **3** | T2N3M0 IVa | 2020/1/14 | - |
| **4** | T4N2M0 IVa | 2019/12/27 | - |
| **5** | T3N3M0 IVa | 2018/3/21 | - |
| **6** | T3N3M0 IVa | 2023/8/15 | 2025/2/25 |
| **7** | T4N2M0 IVa | 2018/11/22 | 2023/12/13 |
| **8** | T4N1M0 IVa | 2020/02/20 | 2023/1/28 |
| **9** | T4N1M0 IVa | 2018/12/15 | 2023/3/13 |
| **10** | T3N3M0 IVa | 2018/07/25 | 2024/10/11 |
